# Neutral-cumulenic diazo (X=C=N_2_) compounds

**DOI:** 10.1039/d6sc04465d

**Published:** 2026-07-02

**Authors:** Sandeep Kumar, Max M. Hansmann

**Affiliations:** a Fakultät für Chemie und Chemische Biologie, Technische Universität Dortmund Otto-Hahn-Str. 6 44227 Dortmund Germany max.hansmann@tu-dortmund.de sandeep.kumar@tu-dortmund.de

## Abstract

Reactive intermediates play a crucial role in organic chemistry, and their isolation and structural characterization have long been of interest to experimental chemists. Among all textbook summaries of reactive intermediates, neutral-cumulenic diazo compounds of the structure X=C=N_2_ (X = R_2_C, R_3_P, R_2_S) are of immense interest due to their unique bonding features and wide synthetic applications in main-group and transition-metal chemistry, even though their direct spectroscopic detection proved highly challenging. In particular, diazoalkenes (R_2_C=C=N_2_) attracted significant interest as synthetically important intermediates and in the late 20th century several research groups probed their existence, mainly indirectly through trapping experiments or directly through matrix-isolation studies. In 2021, the Severin group and our group independently reported the synthesis and characterization of the first room-temperature-stable diazoalkenes, followed by our recent discovery of the diazo heterocumulenes Ph_3_P=C=N_2_ and Ph_2_S=C=N_2_, which initiated a rapidly expanding research field. These bottleable neutral-cumulenic diazo compounds have proven to be pivotal synthons in organic transformations, single carbon atom doping (SCAD) reactions, transition metal chemistry, and low-coordinated main group complexes, with tremendous future promise.

## Abstract 2

Recent advances in the synthesis and applications of stable cumulenic diazo compounds (X=C=N_2_) are highlighted. Their roles in main-group and transition-metal chemistry, as well as C-atom transfer reagents in organic synthesis, are summarized.

## Graphical abstract

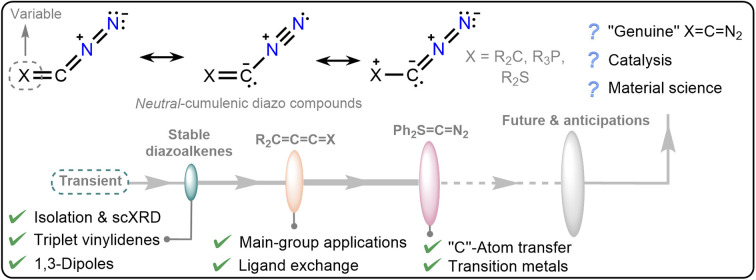

## 1. Introduction

**Fig. 1:**
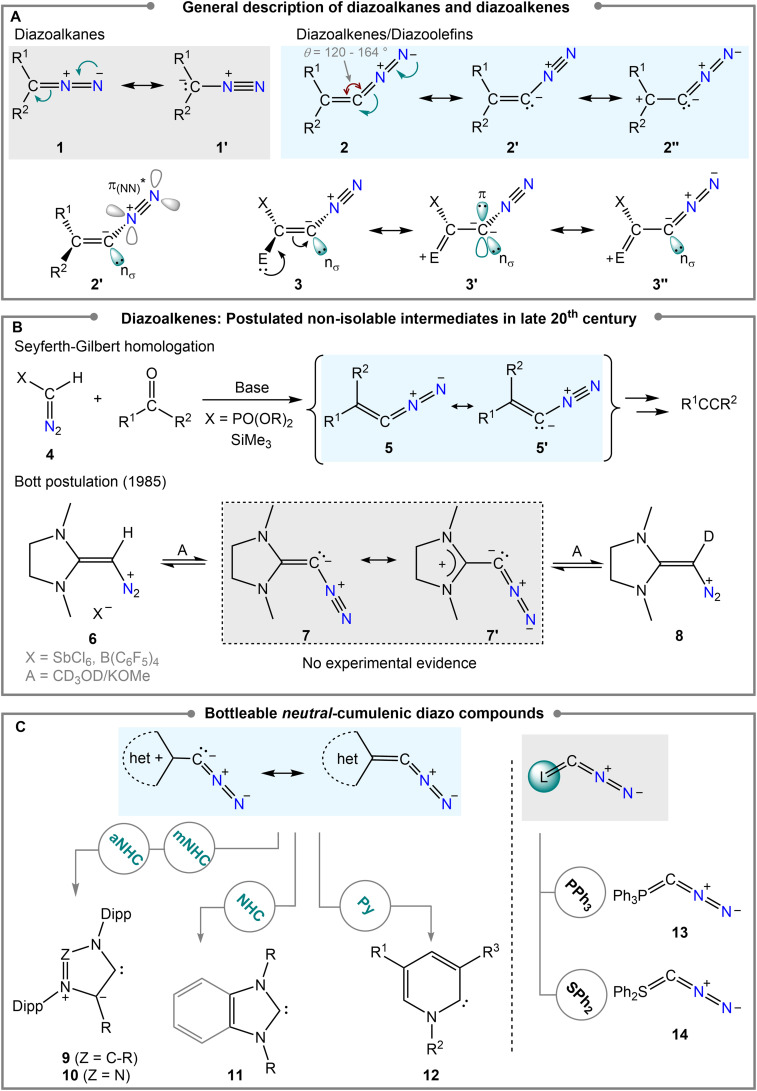
(A) Comparison between diazoalkanes and diazoalkenes; (B) experimental postulation of diazoalkenes as reaction intermediates; (C) examples of bottleable neutral-cumulenic diazo compounds (9–14).

Diazoalkanes of the general formula R^1^R^2^CN_2_ (1/1′, Fig. 1A) have been known for over a century and used as pivotal organic reagents, notably as carbene surrogates and 1,3-dipoles in cycloaddition chemistry.^1–5^ In contrast, the higher unsaturated carbon homologs, the diazoalkenes or diazoolefins (R^1^R^2^C=C=N_2_), are typically considered extremely reactive and unstable, which has caused their chemistry to remain underdeveloped despite their great potential in synthesis.^6^ According to Lewis structure formalism, diazoalkenes can be represented by several Lewis structures with either bent-cumulenic double bonds (2) or as a vinyl anion diazonium cation (2′) or by a C–C polarized Lewis structure containing a C=N=N fragment (2′′) (Fig. 1A).^6^ In standard practice, diazoalkenes are frequently drawn in a linear cumulenic representation; however, based on the quantum chemical investigation of the parent diazoalkene H_2_C=C=N_2_, the linear structure is a transition state on the potential energy hypersurface with an energy magnitude of 23 kcal mol^−1^, higher compared to the global minimum bent structures.^7,8^ The bending at the vinylidene carbon (C^VNL^) perturbs the (C^VNL^)→N_2_ π-back-donation with an in-plane lone-pair (n_σ_) well-localized on the vinylidene carbon (C^VNL^), causing a low dissociation barrier for N_2_ (Δ*E*^‡^_a_ = 6.9 kcal mol^−1^ for H_2_C=CN_2_) from 2′.^7,9^ The inherent lability of diazoalkenes, coupled with the strong thermodynamic driving force for N_2_ extrusion arising from the vinyl diazonium resonance form, has historically limited their isolation and handling. Consequently, low temperature or matrix isolation conditions were employed for diazoalkene detection or trapping. Direct infrared-spectroscopic characterization of difluorodiazoethene F_2_C=C=N_2_ (symmetrical stretching vibration at 2081 cm^−1^) was achieved in a dinitrogen matrix at 11 K.^10,11^ Diazoalkenes, also frequently proposed as fleeting reaction intermediates in organic synthesis, hold immense potential as key intermediates in the Seyferth–Gilbert homologation (5/5′),^12–20^ its Ohira–Bestmann modification,^21–23^ or the Colvin reaction^24–26^ (Fig. 1B). In 1985, Bott investigated the reaction of a resonance-stabilized vinyl diazonium salt (6) with KOMe in CD_3_OD or DMSO-*d*_6_ and observed H/D exchange of the vinylic proton, which led him to conclude that diazoalkene (7/7′) was formed in small amounts in an acid–base equilibrium (Fig. 1b).^27^ However, attempts to synthesize 7 with the stronger base K^*t*^BuO were not successful.^28,29^ Despite spectroscopic characterization in matrix isolation and trapping experiments, the chemistry remained in its infancy until 2021.

In 2021, Severin’s and our research groups independently found that the presence of strongly π-donating substituents (*E*) at the β-carbon atoms (3) leads to an enhanced polarization of the alkene to the vinylic carbon centre (C^VNL^), which adequately stabilizes the diazoalkenes *via* π-electron delocalization towards the diazo moiety, thereby increasing the C=N_2_ double bond character, resulting in an increased bond dissociation energy of the C^VNL^–N_α_ bond (3′′, Fig. 1A). This conceptualization has broadened the scope of synthetically accessible diazoalkenes to include compounds containing (benz)imidazole (9 and 11),^8,30–32^ triazole (10)^33^ and pyridine (12)^34^ scaffolds (Fig. 1C). Interestingly, diazoalkenes could formally also be considered as carbon(0) compounds (carbones)^35–37^ featuring a central C(0) atom flanked by carbene and N_2_ entities (L→C←N_2_). Recent theoretical work by Zhao and co-workers, however, indicates that this view is disfavoured; instead, an EDA-NOCV analysis indicates covalent σ- and π-electron-sharing interactions between the central carbon and its adjacent substituents. Additionally, they support that stabilization of the CN_2_ group arises from in-plane π-conjugation and out-of-plane π-backdonation from carbon to the terminal heteroallene (*vide infra*).^38^ Even though diazoalkenes are not appropriately described as carbon(0) compounds, the formal analysis opens up the prediction of heterodiazo compounds of the structure L→C(N_2_), which was very recently performed computationally by Zhao and Frenking for heteroleptic carbones.^39^ While the unknown homoleptic molecule N_2_CN_2_ was calculated to be thermodynamically unstable, losing N_2_ to form triplet CN_2_ in an exothermic reaction, several heteroleptic combinations were predicted to be stable molecules. In fact, the same stabilization effect for diazoalkenes is expected for diazo heteroalkenes with strongly polarized ylidic element (E)–C^VNL^ π-bonds, which our group could demonstrate using isolable diazo heteroalkenes bearing a triphenyl phosphine (E = P) (13)^40^ and diphenyl sulphide (E = S) (14)^41^ (Fig. 1C). Related to heteroatom-based diazo cumulenes, the seminal work of Bertrand and co-workers in 1987 deserves particular attention. They reported the first evidence for stable cumulenic diazo compounds featuring the connectivity [((^*i*^Pr_2_N)_2_(X)P=C=N_2_; X = Br, Cl], although structural confirmation by X-ray diffraction was not provided.^42–44^ The group also provided the first evidence for [3 + 2] cycloaddition reactivity of P-based diazo cumulenes.

These findings not only advance our understanding of neutral-cumulenic diazo compounds but also open up new possibilities for their practical applications, from small molecule activation to 1,3-dipolar cycloaddition reactants, ligand exchange reactions at the vinylic carbon (C^VNL^) centre, transient vinylidene transfer reagents, and even one-step single carbon atom doping (SCAD) reactions for structural modification of organic molecules. This review will provide a summary of recent advances in the fast-developing chemistry of neutral-cumulenic diazo compounds and their synthetic applications in transition metal, main-group, and SCAD chemistry. Even though anionic-cumulenic diazo derivatives of the structure [E–C=N_2_]M^+^ show similarities to neutral-cumulenic diazo compounds for instance in ligand exchange reactions such as N_2_/CO or N_2_/CNR exchange,^45–48^ they will not be addressed in the present article. Recent independent review articles by Gessner *et al.*^49^ and Liu *et al.*^50^ offer comprehensive analyses of the chronological development and synthetic applications of anionic-cumulenic diazo compounds.

## 2. “Bottleable” neutral-cumulenic diazo compounds: synthesis

### 2.1. Diazo transfer from nitrous oxide (N_2_O)

**Fig. 2:**
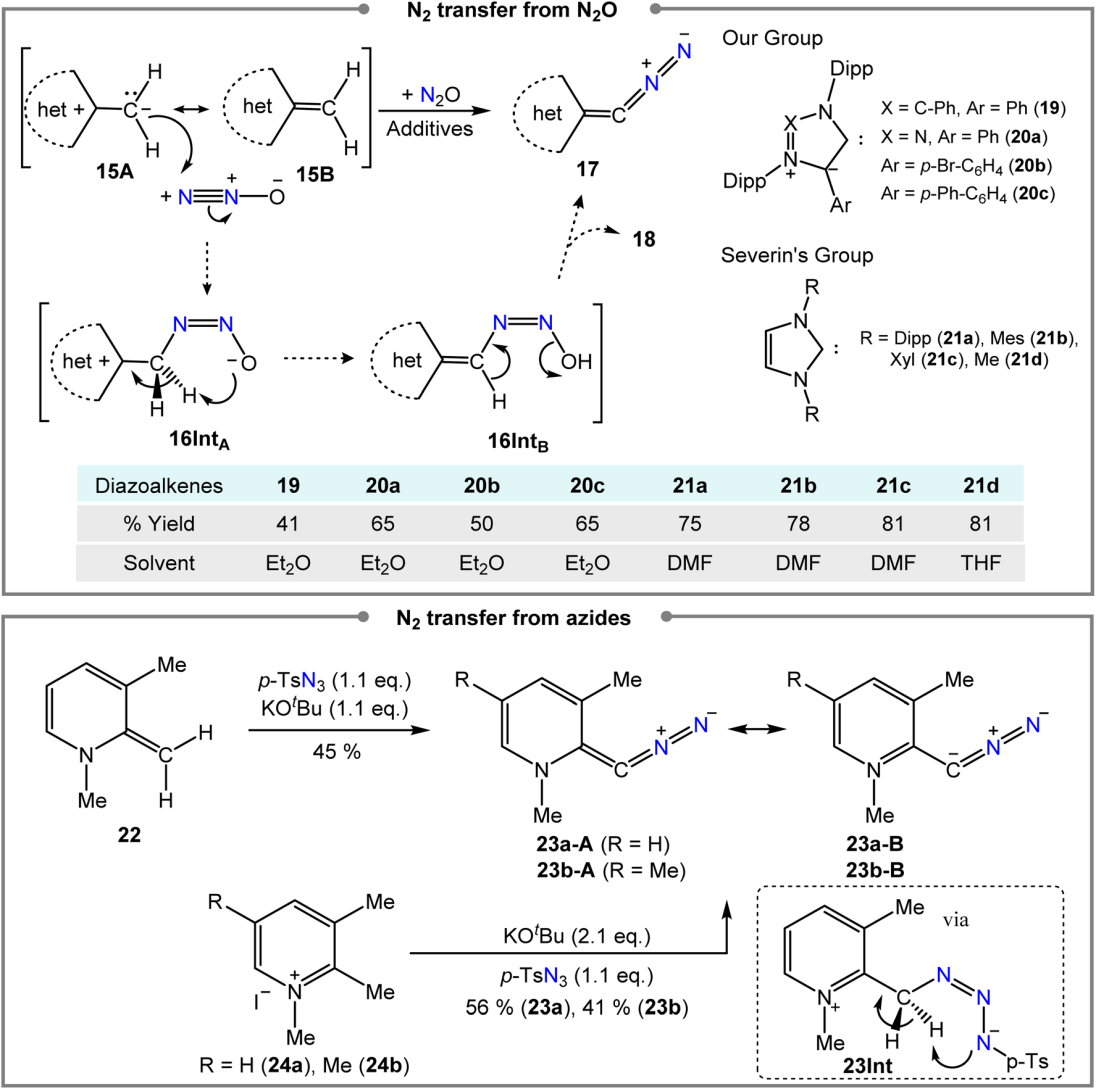
(Top) Room temperature-stable diazoalkenes based on mesoionic/abnormal carbene (19 and 20a–c) and NHC (21a–d) scaffolds obtained from diazo group transfer from N_2_O; (bottom) room temperature stable pyridine-based diazoalkenes (23a–b) obtained from diazo group transfer from *p*-tosyl azide.

Severin *et al.* reported that N-heterocyclic olefins (NHOs)^51–56^ undergo activation of N_2_O in CH_3_CN to afford selectively azo-bridged NHO dimers {NHC(CH)(µ-N_2_)(NHC(CH)} (NHC = IDipp, IMes, and IXyl).^57^ In contrast, our group observed that the more strongly polarized olefins based on mesoionic N-heterocyclic olefins (mNHOs)^58–60^ activate nitrous oxide in Et_2_O to afford the first room temperature stable diazoalkene 19, in which dinitrogen transfer occurs from N_2_O to the olefin.^61^ Upon reacting mNHO with N_2_O, the diazoalkene 19 could be isolated in 41% yield (Fig. 2).^61^ Mechanistically, it is proposed that the electron-rich and highly nucleophilic exocyclic carbon atom^59^ of mNHO adds to N_2_O to give the zwitterionic alkane diazotate (16Int_A_ and 16Int_B_), which, upon dehydration, forms the anticipated diazoalkene. The liberated equivalent amount of H_2_O is scavenged irreversibly by the reaction of a second equivalent of mNHO to give the stoichiometric amide by-product 18.^61^ Our group later observed that this reaction is also applicable to 1,2,3-triazole-derived mNHOs to afford the corresponding stable diazoalkenes (20a–c).^62^ In this case, molecular sieves can be used to trap the formed water, and no stoichiometric by-product is formed, allowing for an isolated yield greater than 50%. In the same year, Severin and co-workers discovered that by changing the solvent to DMF, the dimer formation for the initial described regular NHOs could be suppressed. Instead, the room temperature-stable diazoalkenes (21a–d) were isolated in good yield.^8^ It is noteworthy that Severin *et al.* also reported the synthesis of an anionic diazoalkene in high yield by reacting 21a with ^*n*^BuLi, followed by the addition of BPh_3_ as a trapping agent. The electronic structure of the anionic diazoalkene shows substantial charge concentration on the C^VNL^ and N_terminal_ atoms, and it also shows reactivity accordingly.^63^

### 2.2. Diazo transfer from azides

In 2023, we introduced a new synthetic route to stable diazoalkenes by N_2_ transfer from common, commercially available organic azides.^64^ Activation of nitrous oxide (N_2_O) depends on the nucleophilicity of the employed nucleophile, rendering the pathway only feasible for strong nucleophiles such as mNHOs. Reaction of highly polarised olefins, such as mNHO/NHOs, with *p*-TsN_3_ in the presence of base (KO^*t*^Bu) makes previously discussed diazoalkenes (20a and 21a) readily accessible. This reaction is analogous to the Regitz-diazo transfer,^65^ where a similar intermediate (23Int) resembles the N_2_O addition intermediate (16Int_A_). In fact, it is interesting to highlight that already in 1953 Doering and DePuy wrote in the footnote of their article on the preparation of diazocyclopentadiene about the formal analogy of N_2_-transfer of organometallics with N_2_O and azides.^66^ In the direction of less polarized diazoalkenes or species close to genuine diazoalkenes, N_2_O cannot be activated by pyridine derived olefins 22, which feature significantly reduced nucleophilicity.^67^ Pyridine olefins react with *p*-TsN_3_ in the presence of KO^*t*^Bu to give diazoalkenes 23a/b, featuring a pyridine backbone. The synthesis can also be performed using lutidinium salts (24a/b) with two equivalents of KO^*t*^Bu, avoiding the isolation of the olefin intermediate (Fig. 2).^34^

### 2.3. Ligand/N_2_ exchange

**Fig. 3:**
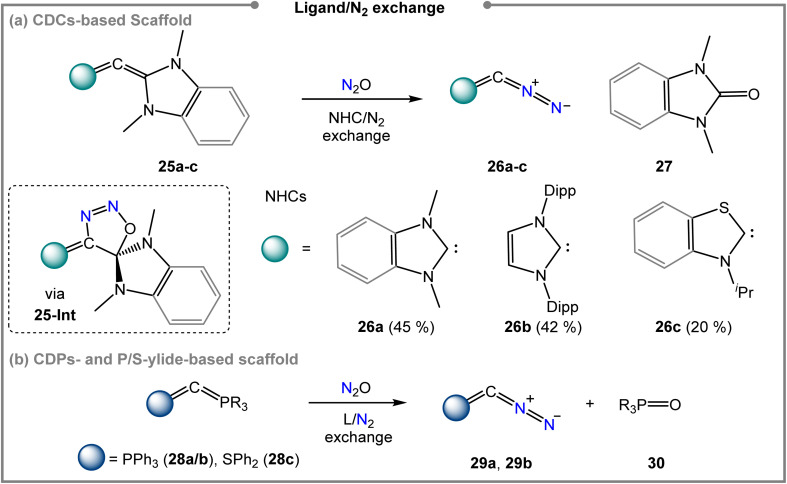
(a) NHC/N_2_ exchange of symmetrical (25a) and unsymmetrical (25b/25c) CDCs to afford NHC-based diazoalkenes (26a–c); (b) diazo transfer to symmetrical (28a, R = PPh_3_) and unsymmetrical (28b, R = P^*n*^Bu_3_) carbodiphosphoranes (CDPs) and P/S-ylide (28c) to afford diazophosphorus (29a) and diazosulphur (29b) ylides.

Frenking, Ong, and co-workers have demonstrated a “hidden π-accepting” character in carbodicarbenes (CDCs)^68,69^ alongside their strong donor abilities.^70^ Specifically, a low-lying, empty π-type orbital centred on the flanking NHC can serve as an acceptor in 1,2-addition reactions, reminiscent of frustrated Lewis pairs. Building on these findings, our group hypothesized that CDCs could react with N_2_O to undergo a [3 + 2] cycloaddition followed by a retro-[3 + 2] cycloaddition, yielding diazoalkenes in a manner analogous to an ozonolysis reaction.^71^ Treatment of various symmetrical and unsymmetrical CDCs (25a–c) with N_2_O results in clean conversion to a range of NHC-based diazoalkenes (26a–c) (Fig. 3) alongside an equimolar by-product: an NHC-based urea derivative 27.^31^ Notably, density functional theory (DFT) calculations support the *Z*-isomer of 26c to be thermodynamically favoured over the *E*-isomer (Δ*G* = + 5.0 kcal mol^−1^). Following the ligand exchange analogy, symmetrical (28a, R = PPh_3_) and unsymmetrical (28b, R = P^*n*^Bu_3_) carbodiphosphoranes (CDPs)^72^ also undergo a similar sequence of [3 + 2]/retro-[3 + 2] cycloaddition with N_2_O, affording the room temperature stable diazophosphorus ylide Ph_3_PCN_2_ (29a) and an equimolar amount of eliminated R_3_PO (30) (R = P^*n*^Bu_3_, PPh_3_).^40^ The P/S ylide Ph_3_P=C=SPh_2_ (28c) also engages in [3 + 2] cycloaddition and elimination reactions with N_2_O to afford selectively the diazosulphur ylide Ph_2_SCN_2_ (29b).^41^

Taken together, these diverse approaches demonstrate how tailored ligand frameworks can stabilize an otherwise highly reactive CN_2_ unit. NHC-based systems (19, 21a–d and 26a–c) generally rely on strong electron donation to the diazo carbon, which promotes efficient charge delocalization and contributes significantly to their high stability. Triazole-derived compounds (20a–c) benefit from additional stabilisation through their mesoionic and conjugative character, whereas pyridine-based systems (23a and 23b) demonstrate that stabilization can also be achieved through a more balanced combination of σ-donor and π-acceptor effects, which renders them the most sensitive compounds. In contrast, phosphorus- (28a) and sulfur-based (28b) frameworks exploit ylidic bonding and polarization effects to support the CN_2_ fragment. Although these strategies differ in their electronic details, they collectively illustrate that the successful stabilization of cumulenic diazo compounds arises from carefully tuned electronic communication between the supporting ligand and the diazo moiety.

## 3. Bonding and structural analysis of diazoalkenes

**Fig. 4:**
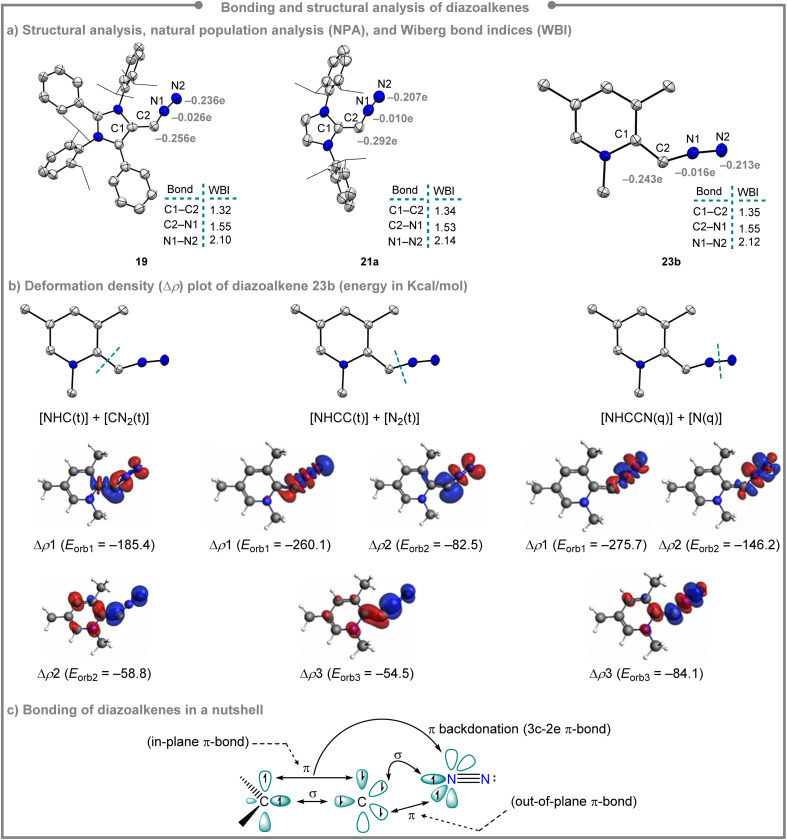
(a) Single-crystal X-ray diffraction (scXRD) structures of the diazoalkenes 19,^32^21a,^8^ and 23b,^34^ together with their calculated natural population analysis (NPA) charges and Wiberg bond indices (WBIs) obtained at the BP86-D3(BJ)/def2-TZVPP level of theory.^38^ Thermal ellipsoids are shown at the 50% probability level. For clarity, the ^*i*^Pr substituents are depicted in wireframe representation; (b) deformation density (Δ*ρ*) plots of the C1–C2, C2–N1, and N1–N2 bonds in 23b, along with the associated orbital interaction energies (Δ*E*_orb_). Charge flow is represented from red to blue, corresponding to electron density redistribution upon bond formation (isovalue = 0.03*e* a.u.^−3^);^38^ reprinted with permission from ref. 38. Copyright 2025 American Chemical Society. (c) Simplified orbital interaction diagram illustrating the bonding interactions in diazoalkenes.^38^

All structurally characterized diazoalkenes exhibit a closely related structural motif and bonding arrangement within the C^carb^–C^VNL^–N1–N2 framework (Fig. 4a). Representative examples, including compounds 19,^32^21a,^8^ and 23b,^34^ exhibit markedly bent C1–C2–N1 bond angles ranging from 122° to 128°, indicating a significant deviation from linearity. The C1–C2 bond lengths (*ca.* 1.40 Å) lie intermediate between those of typical C–C single and double bonds, suggesting substantial π-character. Furthermore, a survey of the Cambridge Structural Database (CSD) of X-ray structures containing terminal CN_2_ moieties reveals that diazoalkenes possess some of the shortest C–N and longest N–N bond lengths reported for this functional group.^6^ Collectively, these structural features demonstrate that diazoalkenes cannot be regarded simply as cumulated olefins and are not adequately described by the classical carbone bonding model. Further insight into the electronic structure of diazoalkenes was provided by Zhao and co-workers through natural population analysis (NPA) and Wiberg bond index (WBI) calculations on 19, 21a, and 23b (Fig. 4a).^38^ In particular, the C2 and N2 atoms bear significant negative charges, averaging −0.267*e* and −0.219*e*, respectively, thereby underscoring the ambident nucleophilic nature of the diazoalkene fragment. The N1–N2 bond exhibits Wiberg bond indices in the range of 2.10–2.14, substantially lower than expected for an idealized N≡N triple bond, indicating pronounced back donation. This finding is in excellent agreement with the experimentally observed red-shifted asymmetric CN_2_ stretching frequencies in the IR spectra.^8,32^ Meanwhile, the C1–C2 and C2–N1 bonds display WBIs of approximately 1.35 and 1.55, respectively, reflecting significant π-delocalization and bonding characteristics intermediate between classical single and double bonds. To further elucidate the bonding in diazoalkenes, EDA-NOCV calculations were performed for 19, 21a and 23b, and the corresponding results are summarized in Table 1.^38^ Across the series, orbital interaction (Δ*E*_orb_) constitutes the dominant attractive component, accounting for approximately 60–70% of the total stabilization energy, thereby indicating a predominantly covalent bonding regime. Detailed NOCV analyses of the representative 23b reveal that the C1–C2 and C2–N1 bonds are primarily formed through electron sharing σ- and π-interactions (Fig. 4b). Notably, the deformation density associated with the C1–N1 bond shows significant π-backdonation from the diazoalkene framework into the vacant π* orbital of the N_2_ fragment. This interaction weakens the N1–N2 bond, accounting for its elongation, reduced bond order, and lower CN_2_ stretching frequencies. Together with the in-plane π-delocalization across the C1–C2–N1–N2 framework, this out-of-plane π-backdonation gives rise to a dual π-delocalization mechanism (Fig. 4c) that governs the electronic structure of reported diazoalkenes. These findings demonstrate that diazoalkenes are best described as π-delocalized heterocumulenes rather than classical carbone analogues.^38^

**Table 1:** Energy decomposition analysis combined with natural orbitals for chemical valence (EDA-NOCV) results for the discussed diazoalkenes 19, 21a, and 23b, calculated at the BP86-D3(BJ)/TZ2P level of theory using fragments in their electronic triplet (t) and quartet (q) states. The dispersion energy contribution (Δ*E*_disp_) is omitted from the table owing to its negligible contribution to the overall interaction energy. The data shown were adapted from ref. 38

Fragments	Δ*E*_int_ (kcal mol^−1^)			Δ*E*_Pauli_ (kcal mol^−1^)			Δ*E*_elstat_ (kcal mol^−1^)			Δ*E*_orb_ (kcal mol^−1^		
	19	21a	23b	19	21a	23b	19	21a	23b	19	21a	23b
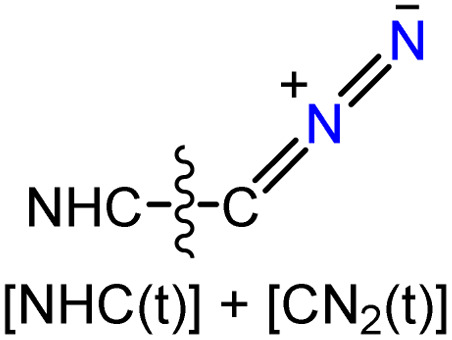	−232.3	−247.6	−217.0	343.2	252.0	256.9	−215.5	−178.8	−182.7	−349.8	−309.6	−285.7
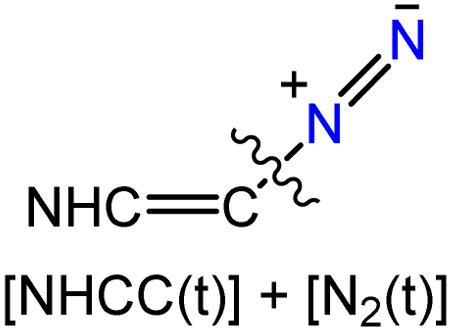	−252.3	−253.7	−245.0	388.8	379.6	381.4	−199.6	−196.3	−197.1	−435.8	−431.4	−426.4
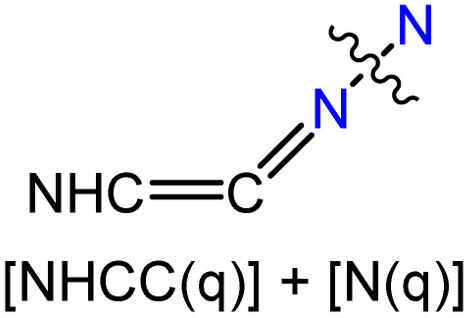	n.d.^a^	n.d.	−241.0	n.d.	n.d.	545.2	n.d.	n.d.	−237.4	n.d.	n.d.	−547.2

a n.d. = not determined.

## 4. Reactivity of neutral-cumulenic diazo compounds

### 4.1. Diazoalkenes: versatile reagents for cycloaddition chemistry

**Fig. 5:**
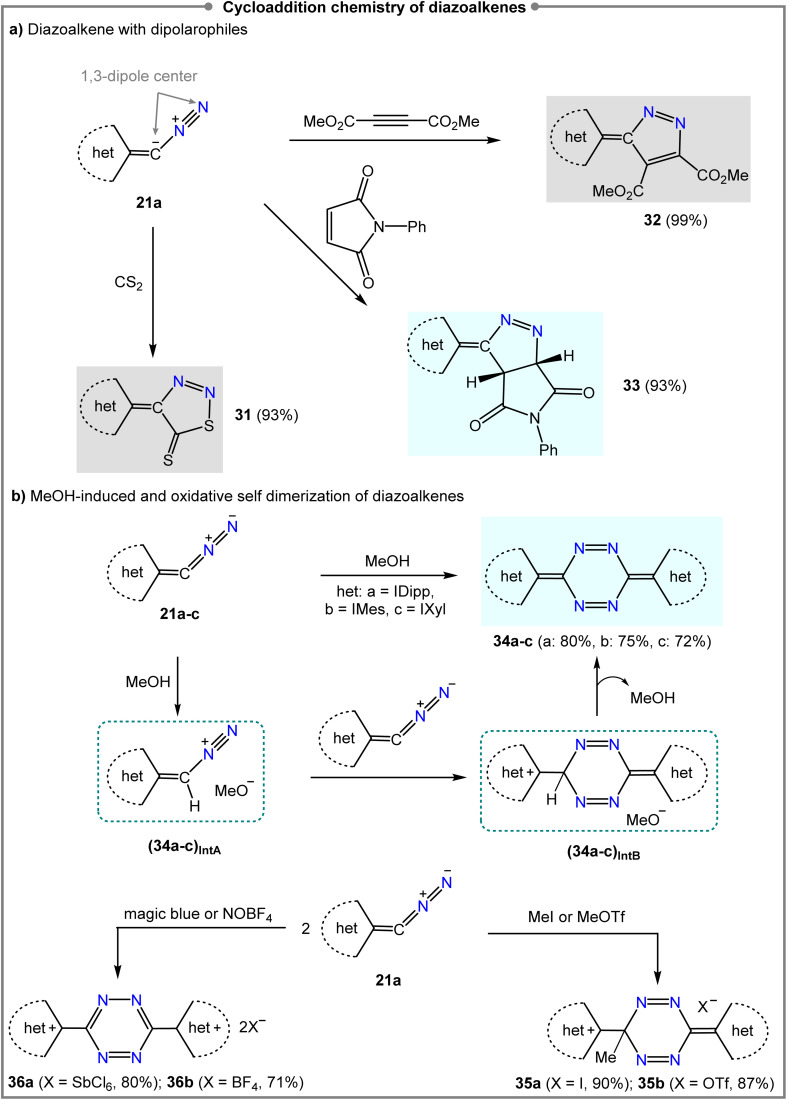
(a) 1,3-Dipolar cycloaddition reactions of diazoalkene 21a; (b) head-to-tail (htt) dimerization of N-heterocyclic diazoalkenes.

Diazoalkanes (1, Fig. 1a) readily undergo [3 + 2] cycloaddition reactions with a wide range of dipolarophiles, and these 1,3-dipolar cycloaddition (Huisgen's cycloaddition) reactions have emerged as a fundamentally important synthetic strategy in contemporary heterocyclic chemistry.^73,74^ Resonance-stabilised diazoalkenes can react as 1,3-dipoles similar to diazoalkenes. In the reaction of 21a with polarized dienophiles such as carbon disulphide, dimethyl acetylene dicarboxylate (DMAD) and *N*-phenylmaleimide, the corresponding cycloaddition products 31, 32 and 33 were isolated in excellent yields (Fig. 5a).^8^

Intriguingly, N-heterocyclic diazoalkenes 21a–c undergo methanol-induced head-to-tail (htt) dimerization, formally representing a [3 + 3] cycloaddition *via* the CN_2_ group, to afford quinoidal tetrazines 34a–c in moderate to excellent yields (Fig. 5b).^75^ Mechanistically, the formation of quinoidal tetrazines 34a–c can be rationalized by initial protonation of diazoalkenes 21a–c at the ylidic exocyclic carbon (C^VNL^) atom by MeOH, generating vinyl diazonium salt (34a–c)_IntA_. The subsequent reaction with a second equivalent of diazoalkene affords an intermediate of type (34a–c)_IntB_, which upon elimination of solvent (MeOH) yields the corresponding products. Additionally, indirect evidence for the proposed intermediates of type (34a–c)_IntB_ was obtained through the synthesis of the structural analogues 35a and 35b (Fig. 5b).^75^ These compounds were prepared by reacting the corresponding diazoalkene 21a with 0.5 equivalents of MeI or MeOTf, respectively. Severin and co-workers also observed the oxidation-induced dimerization of diazoalkene 21a. The diazoalkene bearing Dipp wing-tip groups was converted into tetrazine salts 36a and 36b upon reaction with either “Magic Blue” or NOBF_4_ (Fig. 5b). The structural identity of the dimerization products was unambiguously confirmed by single-crystal X-ray diffraction (scXRD) analysis.^75^

### 4.2. Neutral-ligand exchange at a “C(0)” centre in diazoalkenes

**Fig. 6:**
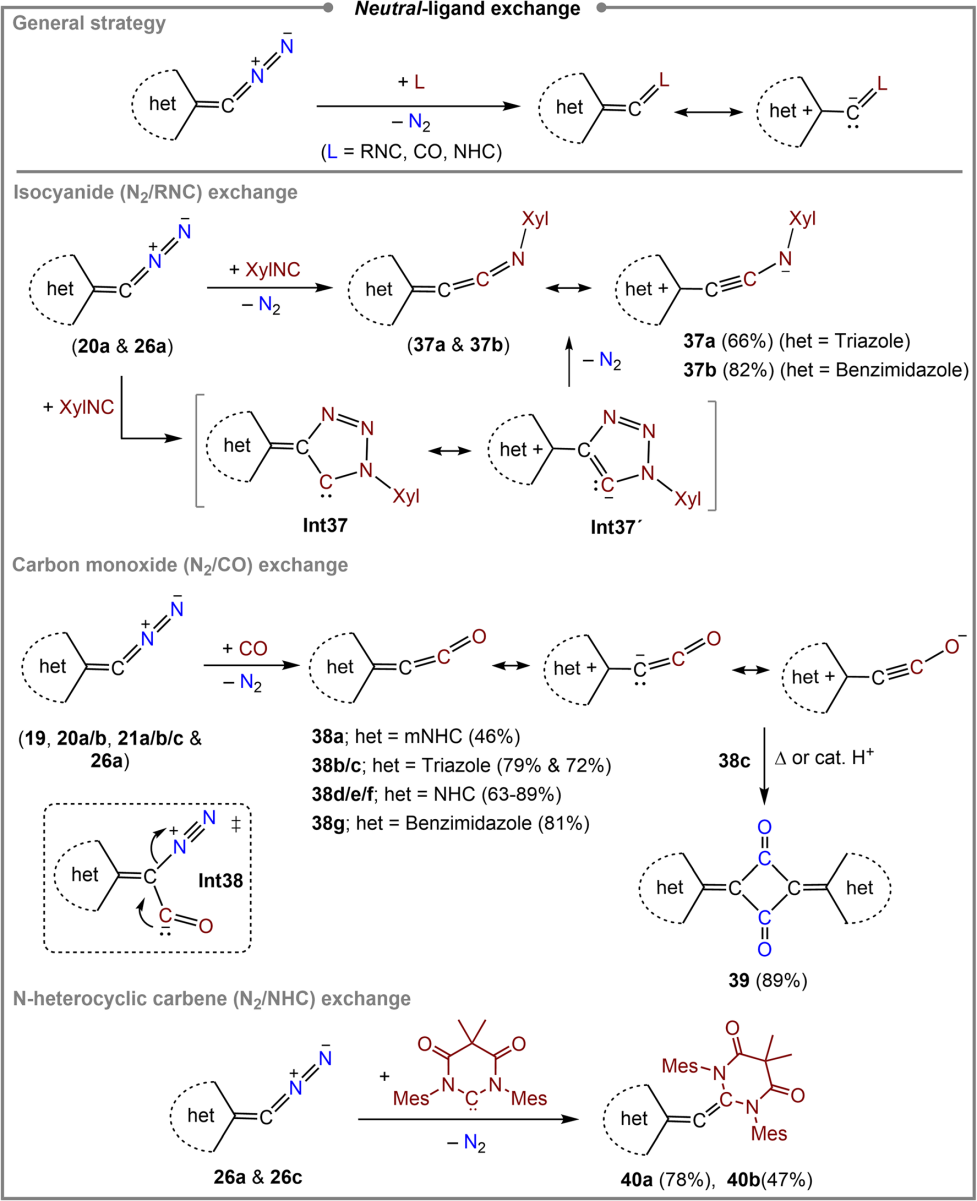
Neutral-ligand exchange reactions at a vinylidene carbon centre.

The reaction of the discussed stable diazoalkenes with ambiphilic neutral ligands, including isocyanides (RNC), carbon monoxide (CO), and N-heterocyclic carbenes, promotes displacement of N_2_ through formal ligand substitution pathways. These transformations provide access to heterocumulenic frameworks such as vinylidene ketenimines (37a & 37b)^31,62^ and vinylidene ketenes (38a–g),^31,62,76^ and heteroleptic carbodicarbenes (40)^31^ (Fig. 6). Notably, mechanistically RNC ligand exchange proceeds through stepwise [3 + 2] cycloaddition to afford intermediate Int37/Int37′, followed by a [3 + 2] cycloreversion under release of dinitrogen (Fig. 6a).^62^ In contrast, in the case of CO, a concerted mechanism occurred, in which the diazoalkenes add to the LUMO of CO, followed by a concerted backflip of electron density *via* transition state Int38 (Fig. 6b).^62^ Severin and co-workers further demonstrated the feasibility of N_2_/CO exchange reactions using imidazole-derived diazoalkenes 21a/b/d, leading to the formation of vinylidene ketenes 38d–f (Fig. 6b),^76^ which exhibited high thermal stability. Upon thermal activation or treatment with catalytic amounts of acid, these species underwent conversion to the corresponding dimeric structure 39.^76^ In a related study, benzimidazole-derived diazoalkenes 26a and 26c were found to readily participate in N_2_ displacement by N-heterocyclic carbenes [*e.g.*, diamidocarbenes (DAC)], furnishing the corresponding heteroleptic carbodicarbenes 40a and 40b in moderate to good isolated yields, respectively (Fig. 6c).^31^

#### 4.2.1. Comparative assessment of diazoalkenes, carbones, vinylidene ketenes, and vinylidene ketenimines

**Fig. 7:**
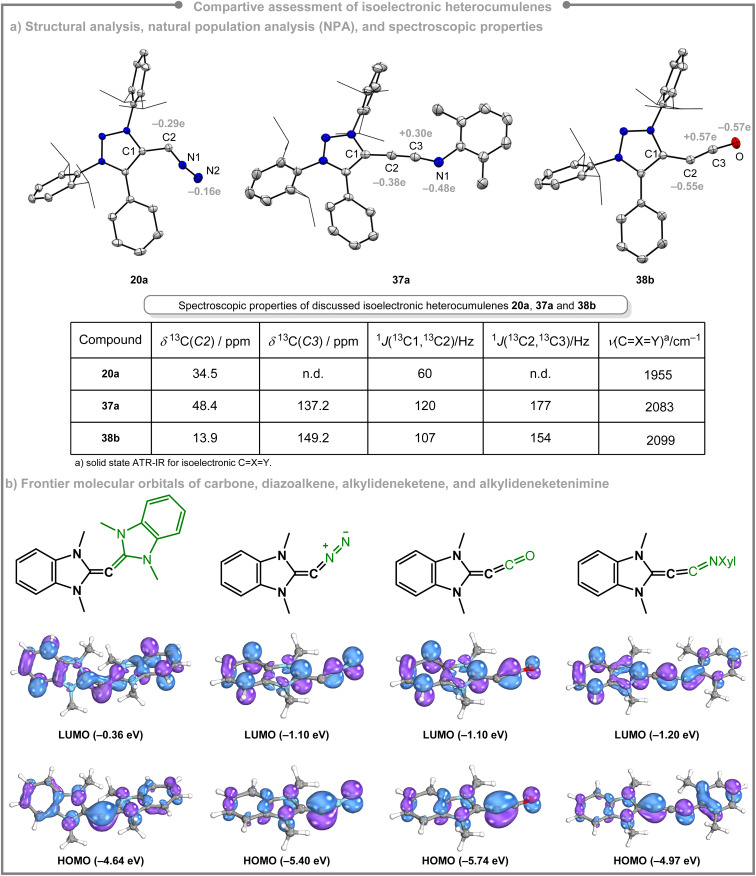
(a) Single-crystal X-ray diffraction (scXRD) structures of the diazoalkene 20a,^62^ alkylidene ketenimine 37a,^62^ and alkylidene ketene 38b,^62^ together with their calculated natural population analysis (NPA) charges (calculations were performed on simplified systems; see ref. 62) obtained at the BP86-D3(BJ)/def2-TZVPP level of theory. Thermal ellipsoids are shown at the 50% probability level. For clarity, the ^*i*^Pr substituents are depicted in a wireframe representation; (b) frontier molecular orbitals (HOMO & LUMO) of the bent carbodicarbene (carbone) and the isoelectronic series R_2_C=C=X (X=N_2_ (26a),^31^ CO (38g),^31^ and CNXyl (37b)^31^) obtained at the PBE0-D3(BJ)/def2-TZVP level of theory.

From an electronic-structure perspective, diazoalkenes (19–26c), vinylidene ketenes (38a–g), and vinylidene ketenimines (37a & 37b) constitute an isoelectronic heterocumulene series, whereas carbones (L_1_CL_2_; L_1_ and L_2_ = neutral 2e^−^ donor ligands)^36,37^ represent the limiting case of carbon-centered electron localization. In diazoalkenes (for instance 26a), the HOMO is primarily a π-type orbital delocalized over the C=C=N_2_ fragment (Fig. 7b), while the HOMO−1 possesses significant σ-type lone-pair character at the α-carbon.^31^ This orbital arrangement accounts for their pronounced nucleophilicity and partial resemblance to carbones. However, unlike genuine carbones, the occupied orbitals remain strongly coupled to the delocalized π-system and the LUMO corresponds to the antibonding π* orbital of the cumulene framework (Fig. 7b). Structurally, this electronic situation is reflected in a markedly bent geometry, with a C1–C2–N angle of approximately 122° and a relatively small ^1^*J*(^13^C1–^13^C2) coupling constant of *ca.* 60 Hz (for instance 20a,^62^Fig. 7a), consistent with substantial sp^2^ character at C1 (C^VNL^). Upon replacement of N_2_ by CO, vinylidene ketenes (38a–g) exhibit greater cumulenic delocalization. This is manifested by widening of the C1–C2–C3 angle to *ca.* 146°, shortening of the C2–C3 bond to about 1.237 Å, and an increase of ^1^*J*(^13^C1–^13^C2) to approximately 107 Hz (38b,^62^Fig. 7a). The HOMO becomes more extensively delocalized over the C=C=C=O framework, while the LUMO retains cumulene π*-character (Fig. 7b). Vinylidene ketenimines (37a,^62^ & 37b,^31^) display the highest degree of cumulenic character within the series. Their nearly linear geometry (C1–C2–C3 ≈ 176°), short C2–C3 bond length (≈1.233 Å), and large coupling constants [^1^*J*(^13^C1–^13^C2) ≈ 120 Hz and ^1^*J*(^13^C2–^13^C3) ≈ 177 Hz] (37a) indicate substantial sp-hybridization and efficient π-delocalization across the entire framework (Fig. 7a). Consequently, both structural and spectroscopic parameters support increasing cumulenic character in the order: diazoalkene < vinylidene ketene < vinylidene ketenimine. In contrast, carbones are best described as carbon(0) species of the form L→C←L, where the HOMO and HOMO−1 (Fig. 7b) correspond to two genuine nonbonding orbitals (σ and π lone pairs) centered on the central carbon.^36,37,77^ Their characteristic bent L–C–L geometries (typically 120–150° depending on the donor ligands) arise from localization of electron density at carbon rather than from a delocalized cumulene π-system. Thus, while diazoalkenes share with carbones the presence of occupied σ- and π-type orbitals at carbon, their bond lengths, bond angles, coupling constants, and frontier orbital distributions clearly place them closer to polarized heterocumulenes than to true carbon(0) compounds.

### 4.3. Diazoalkenes in main-group chemistry

#### 4.3.1. Group 13

**Fig. 8:**
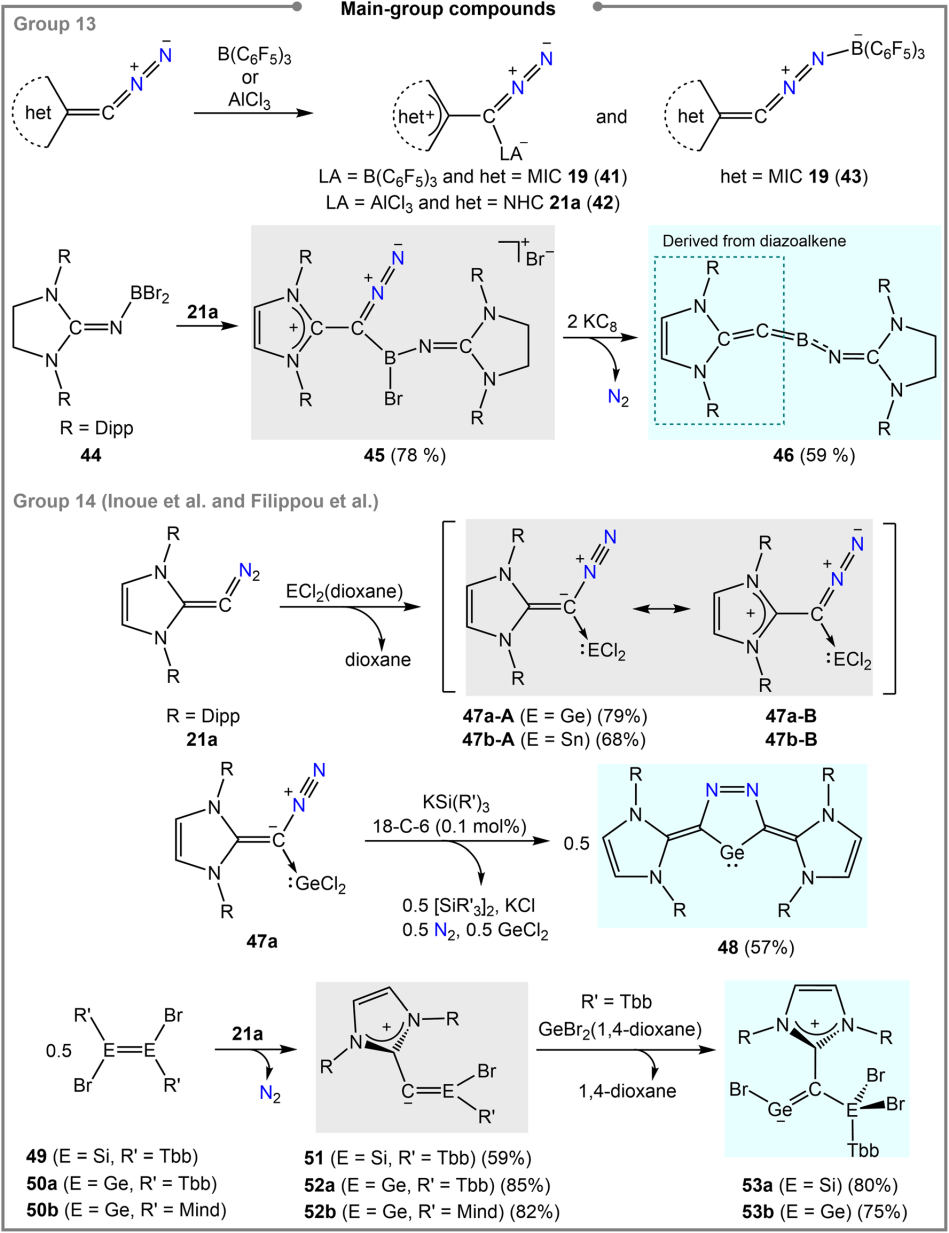
Reactivity of group 13 and 14 complexes towards diazoalkenes to access novel low-coordinate structural motifs.

The aforementioned computational analysis of diazoalkenes shows pronounced negative natural charges of the CN_2_ fragment at C^VNL^ [−0.26e^−^ (19),^32^ −0.35e^−^ (21a)^8^] and N_term_ [−0.20e^−^ (19),^32^ −0.10e^−^ (21a)^8^], enabling diazoalkenes to be potent nucleophiles to coordinate electrophiles either at the vinylic carbon (C^VNL^) or terminal nitrogen atoms (N_term_). In the reaction with the main group 13 Lewis acids [B(C_5_F_5_)_3_ and AlCl_3_], both possible Lewis acid/base adducts (41, 42, and 43) could be isolated and structurally confirmed by sc-XRD (Fig. 8).^8,61^ The regioselectivity of the borane adduct formation can be altered by the choice of solvent and reaction conditions: toluene at room temperature yields a selective formation of 41, while Et_2_O at −40 °C gives exclusive access to 43.^61^

Organic cumulenes are textbook compounds, containing a linear carbon chain skeleton and three or more consecutive C=C double bonds.^78–81^ Cumulenes possess multidirectional applications, extending their reach in semiconductors and the molecular wires industry, as well as showing potential use as models for the carbon allotrope carbynes.^82–87^ BN and CC fragments are correlated *via* the isolobal analogy, and it was anticipated that BN-embedded conjugated systems like cumulenes offer versatility in applications due to alteration in frontier orbital energies and modulated electronic structures.^88,89^ BN-doped polycyclic aromatic hydrocarbons (PAHs) have been reported;^90^ however, experimentally validated examples of metal-free acyclic BN-embedded [*n*]cumulenes (*n* ≥ 3 and *n* represents the number of cumulated double bonds) remain scarce.^91^ Recently, Mo, Xu and co-workers isolated hybrid heterocumulenes utilizing the versatility of diazoalkenes. An N-heterocyclic imino (NHI)-substituted dibromoborane (44) undergoes facile displacement of the bromine atom with (IDipp)CN_2_ (21a) to afford a rare α-boryl diazo compound 45 as a yellow solid in 78% yield (Fig. 8). 2e^−^ reduction of 45 with KC_8_ afforded a thermally robust BN-hybrid heterocumulene 46 as an air and moisture-sensitive orange solid (Fig. 8). Single-crystal X-ray diffraction analysis (sc-XRD) reveals a bent CCBNC chain, and the bending could be attributed to the resonance of the diamino-capped group, which imparts partial lone-pair character at vinylic carbon (C^VNL^) and reduces π-backbonding to the NHC backbone.^91^

#### 4.3.2. Group 14

Contemporary main group chemistry has experienced a renaissance in isolating low-coordinate main group compounds by utilizing structurally related ligands, namely N-heterocyclic imines (NHIs),^56,92^*N*-heterocyclic olefins (NHOs),^51–56^ and N-heterocyclic carbenes (NHCs). For instance, NHC-adducts of dihalotetrylenes EX_2_ (E = Si–Sn; X = Cl, Br) have been isolated due to their electron-donating capabilities, combined with kinetic stabilization provided by the substituents at the nitrogen atom of NHCs. Additionally, NHOs have also been proven to be excellent donors due to their nucleophilic and ylidic character on the exocyclic carbon centre, therefore standing in a very close relationship to the discussed diazoalkenes. The research group of Inoue utilized the nucleophilicity of the vinylic carbon (C^VNL^) to synthesize the ECl_2_ (E = Ge, Sn) Lewis acid–base adduct of diazoalkenes. To synthesize Ge complex 47a and Sn complex 47b (Fig. 8),^93^ the respective (dioxane)tetrylene dichlorides were treated with a suspension of diazoalkene 21a, and the anticipated adducts could be isolated in good yields by removing the solvent. The IR stretching frequency corresponding to N_2_ in adducts 47a and 47b exhibits an intense absorption band at 2039 cm^−1^, which is higher than that of the non-coordinated diazoalkene 21a (1984 cm^−1^). Notably, light-mediated dinitrogen liberation from adducts 47a and 47b with light in the wavelength range of 300–360 nm results in unselective decomposition. However, treatment of 47a with potassium hypersilanides {KSiR_3_; R = SiMe_3_, (SiMe_3_)_2_Si(^*i*^Pr)_3_, (SiMe_3_)_3_Si(tolyl)_3_} along with a catalytic amount of 18-crown-6 afforded the cyclic bis-vinyl germylene 48 (Fig. 8).^93^ Single-crystal X-ray diffraction (scXRD) and quantum chemical calculations of 48 revealed an extensive 6π-electron delocalization in the five-membered germylene core Ge(C^VNL^)_2_N_2_. This is also further strengthened by the calculated nuclear independent chemical shift (NICS) values for 48. NICS(1) and NICS(−1) values of −9.1 and −8.7 ppm, as well as the NICS(1)_*zz*_ and NICS(−1)_*zz*_ values of −18.1 and −18.6 ppm, are indicative of a strong diatropic ring current, as benzene diatropic ring current shows a value of −10.1 and −28.8 ppm for NICS(1) and NICS(1)_*zz*_ at the same level of theory.^93^

Vinylidene (:C=CR_2_) is a transient, unsaturated carbene and readily tautomerizes to the respective alkyne (RC≡CR). Yet, this instability is likely its most utilized trait, with the Fritsch–Buttenberg–Wiechell (FBW) rearrangement,^94–97^ being a key step in both the Corey–Fuchs reaction^98^ and the Seyferth–Gilbert homologation.^18^ While vinylidenes can only be isolated in frozen argon matrices,^99,100^ their stable transition metal complexes [L_*n*_M=C=CR_2_], containing an electrophilic α-carbon and nucleophilic β-carbon atom, have been extensively studied in a variety of stoichiometric and catalytic transformations.^101–104^ Notably, the carbon and heavier analogues, mixed vinylidenes—2-tetrelavinylidene (:C=ER_2_) and 1-tetrelavinylidene^105–107^ (:E=CR_2_); E=Si–Pb)—are scarce in the literature and this could be interpreted by the quantum chemical calculations of the potential energy surface (PES) having one tetrel and carbon atom, and two hydrogen atoms, which revealed that 2-tetrelavinylidenes (C=EH_2_) are the least stable isomers, isomerizing easily *via* the *trans*-bent tetrelaacetylenes HE≡CH to the thermodynamically most stable 1-tetrelavinylidenes (E=CH_2_).^108–111^ In the pursuit of isolating some of these isomer derivatives under standard laboratory conditions, Filippou and co-workers selected diazoalkenes as potential reagents for vinylidene transfer to tetrel(II) centres. A straightforward reaction between diazoalkene 21a and (*E*)-R′BrE=EBrR′ (R′ = Tbb, Mind; E = Si, Ge) (49, 50a and 50b) afforded selective conversion to unprecedented 2-tetrelavinylidenes (*Z*)-(NHC)C=E(Br)R′ (51, 52a and 52b) in moderate to good yield (Fig. 8).^112^ All 2-tetrelavinylidenes 51, 52a and 52b have a planar vinylidene core, a highly bent-dicoordinated vinylidene carbon atom (C^VNL^), a very short E=C^VNL^ bond, and an almost orthogonal orientation of the NHC five-membered ring to the vinylidene core. Quantum chemical calculations of the electronic structures of 51 and 52a suggest a significantly bent-1-tetrelaallene and tetrelyne character. Furthermore, the synthetic potential of 2-tetrelavinylidenes is demonstrated by the synthesis of the unprecedented NHC-supported bromogermyne BrGe=C(EBr_2_Tbb)(NHC) (53a (E = Si) and 53b (E = Ge)) (Fig. 8).^112^

#### 4.3.3. Group 15

**Fig. 9:**
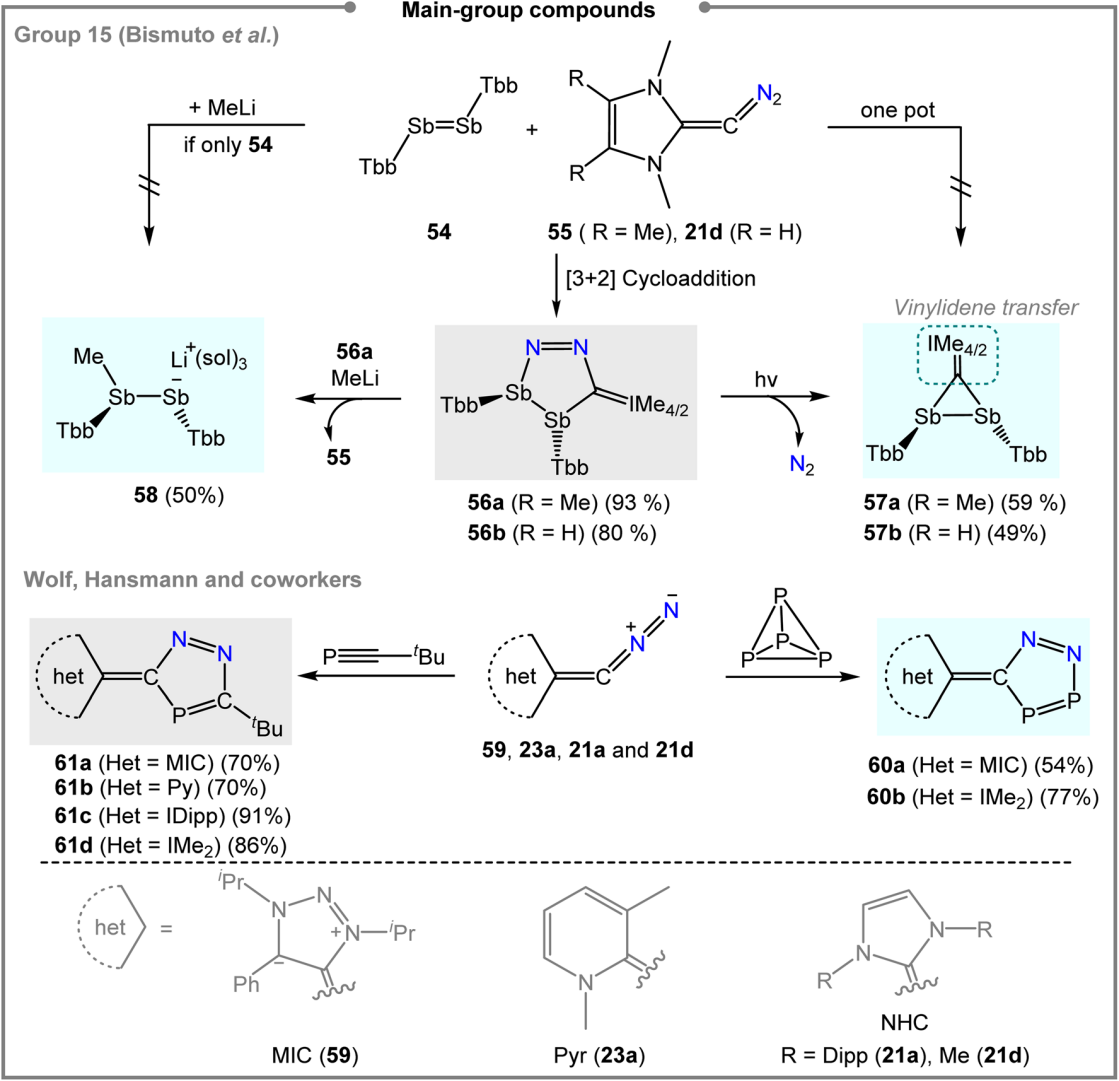
Reactivity of pnictogens towards diazoalkenes to access novel main-group complexes.

A very common approach to access organic N-heterocycles involves the 1,3-dipolar cycloaddition reaction of diazonium betaines (diazoalkanes, azides, and nitrile oxides) with an unsaturated skeleton.^113^ The Huisgen dipolar [3 + 2]-cycloaddition reaction between an azide and an alkyne to afford 1,2,3-triazole rings possesses significant potential in the discussed domain^74^ and also governs the foundational work for “Click Chemistry”.^114^ However, inorganic heterocycles containing heavier pnictogens in the core skeleton rings are scarce, presumably due to the inert lone-pair and the unreactive nature of the π-bond in heavier dipnictenes for functionalization. The sterically protected distibene π-bond undergoes a formal [3 + 2]-cycloaddition with a nitrile oxide (class of diazobetaines) to afford a non-planar 1-oxa-5-aza-2,3-distibacyclopent-4-ene derivative as reported by Tokitoh *et al.*^115^ Recently, the Bismuto group anticipated that sterically deshielded diazoalkenes could also engage in a formal [3 + 2]-cycloaddition reaction with the sterically shielded heavier dipnictene π-bond. Diazoalkenes 21d and 55 undergo a formal [3 + 2]-cycloaddition with a distibene (54) to afford rare 5-membered diazadistiboylidenes 56a and 56b under ambient conditions, with yields of 93% and 80%, respectively (Fig. 9).^116^

56a shows a dynamic equilibrium with a non-coordinated diazoalkene (55) and free distibene, which was also verified experimentally, as it shows only diazoalkene-type reactivity and also allows switching of the diazoalkene with 21d to afford diazadistiboylidenes 56b. 5-Membered diazadistiboylidenes 56a and 56b undergo light-driven N_2_ deletion to selectively afford unprecedented three-membered methylenedistibiranes 57a and 57b. A stepwise synthetic pathway to methylenedistibiranes could be interpreted as the transfer of the N-heterocyclic vinylidene moiety^117^ from diazoalkenes to distibenes. Ring opening of 56a to afford an acyclic motif could be facilitated by reacting it with an alkyl lithium reagent (*e.g.* MeLi) to synthesize a rare lithium cation separated diantimonide 58. It is worth mentioning that the direct lithiation of distibenes with alkyl lithium reagents leads to a complicated mixture of products, highlighting the application of diazoalkenes in isolating novel structural motifs.^116^

As demonstrated earlier, diazoalkenes undergo cycloaddition reactions with polar unsaturated organic molecules. Similarly, the joined work of the Wolf and our group could show that diazoalkenes (21a, 21d, 23a, and 59) readily react with *tert*-butylphosphaalkyne (^*t*^BuCP) and white phosphorus (P_4_) to afford novel phosphorus-containing heterocycles, namely 3*H*-1,2,4-diazamonophospholes 61a–d and 1,2,3,4-diazadiphospholes 60a and 60b (Fig. 9).^118^

### 4.4. Neutral-cumulenic diazo compounds in transition metal TM chemistry

**Fig. 10:**
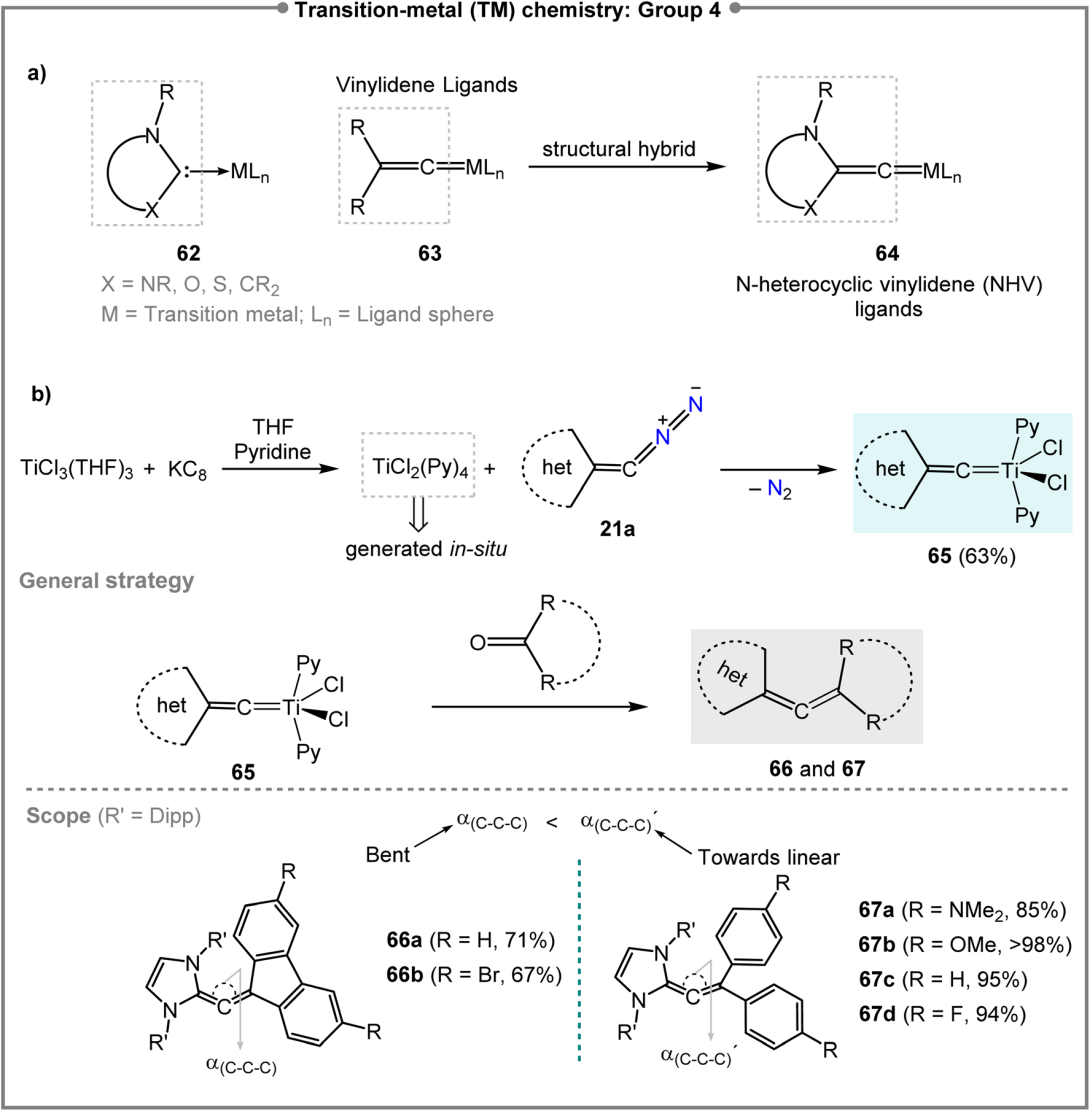
(a) NHV ligands (64) obtained by a formal structural merge between NHCs and vinylidene ligands; (b) synthesis of a Ti-alkylidene complex (65) and its reactivity with ketones to afford bent N-heterocyclic allenes.

N-heterocyclic carbenes (NHCs) are widely considered as effective carbon/σ-donor ligands and are crucial in transition-metal (TM) catalysis,^119^ while vinylidenes also play a significant role in numerous TM-catalysed transformations.^120–123^ The combination of these two entities yields N-heterocyclic vinylidenes (NHVs) 64 (Fig. 10a), a promising yet largely underexplored class of ligands.

Diazo compounds (1/1′; Fig. 1a) are suitable precursors for carbene complexes;^124^ however, the synthesis of vinylidene complexes from diazo compounds is uncommon^125^ due to the facile elimination of N_2_ at low temperatures.^97^ In this context, isolable and thermally robust neutral-cumulenic diazo compounds, such as diazoalkenes, may serve as convenient sources of NHVs. Note that while there are stepwise synthetic strategies to access NHV ligands starting from NHOs by two-fold deprotonation sequences,^117^ diazoalkenes are ideal precursors since only N_2_ liberation is required. The exocyclic carbon atom in diazoalkenes exhibits pronounced ylidic character, which facilitates the formation of stable Lewis acid–base adducts with metal complexes such as AuCl, RhCl(CO)_2_, PdCl(allyl), and Cr(CO)_5_, in which the diazo moiety remains intact.^6,126^ Transition metal (TM) complexes bearing N-heterocyclic vinylidene ligands have been well documented in the mini-review reported by Severin and co-workers.^127^

#### 4.4.1. Group 4

Allenes of the general formula R_2_C=C=CR_2_ (R = hydrogen, alkyl, or aryl) contain two orthogonal π-bonds, resulting in a linear geometry about the central carbon atom (C^cent^), with the terminal substituent planes oriented mutually perpendicular to each other.^128^ However, the last two decades have witnessed significant progress toward bent-allenes,^68^ where strong electron-donating capping groups at the terminal carbon (C^term^) atoms give rise to pronounced ylidic character and induce angle bending at the central carbon atom (C^cent^).^77^ Conceptually, metal-vinylidene complexes (L_*n*_M=C=CR_2_; L_*n*_ = ligand sphere) may serve as versatile intermediates for the facile synthesis of structurally diverse bent or linear allenes. This conceptual prediction was promptly validated as Severin and co-workers reported that the *in situ* generated Ti(ii) complex TiCl_2_(py)_2_ (py = pyridine), upon reaction with diazoalkene 21a in THF, provides facile access to the unprecedented Ti-alkylidene complex 65 (Fig. 10b).^129^ The complex 65 exhibits a distorted square-pyramidal coordination geometry at the Ti-centre, featuring two axially oriented pyridine ligands and two equatorial chloro ligands along with an N-heterocyclic vinylidene (NHV) motif. Structurally characterized 65 undergoes metathesis reaction with cyclic (fluorene-based) and acyclic (diaryl-based) ketones to afford targeted bent N-heterocyclic allenes (66 and 67) in good to excellent yield (Fig. 10b). Notably, the push–pull allenes with fluorenylidene capping groups (66a and 66b) revealed markedly bent C=C=C units (*α*_(C–C–C)_ < 140°), whereas the less polarized diarylmethylidene analogues (67a–d) were found to display more linear geometry (*α*_(C–C–C)′_ > 150°).^129^

#### 4.4.2. Group 5

**Fig. 11:**
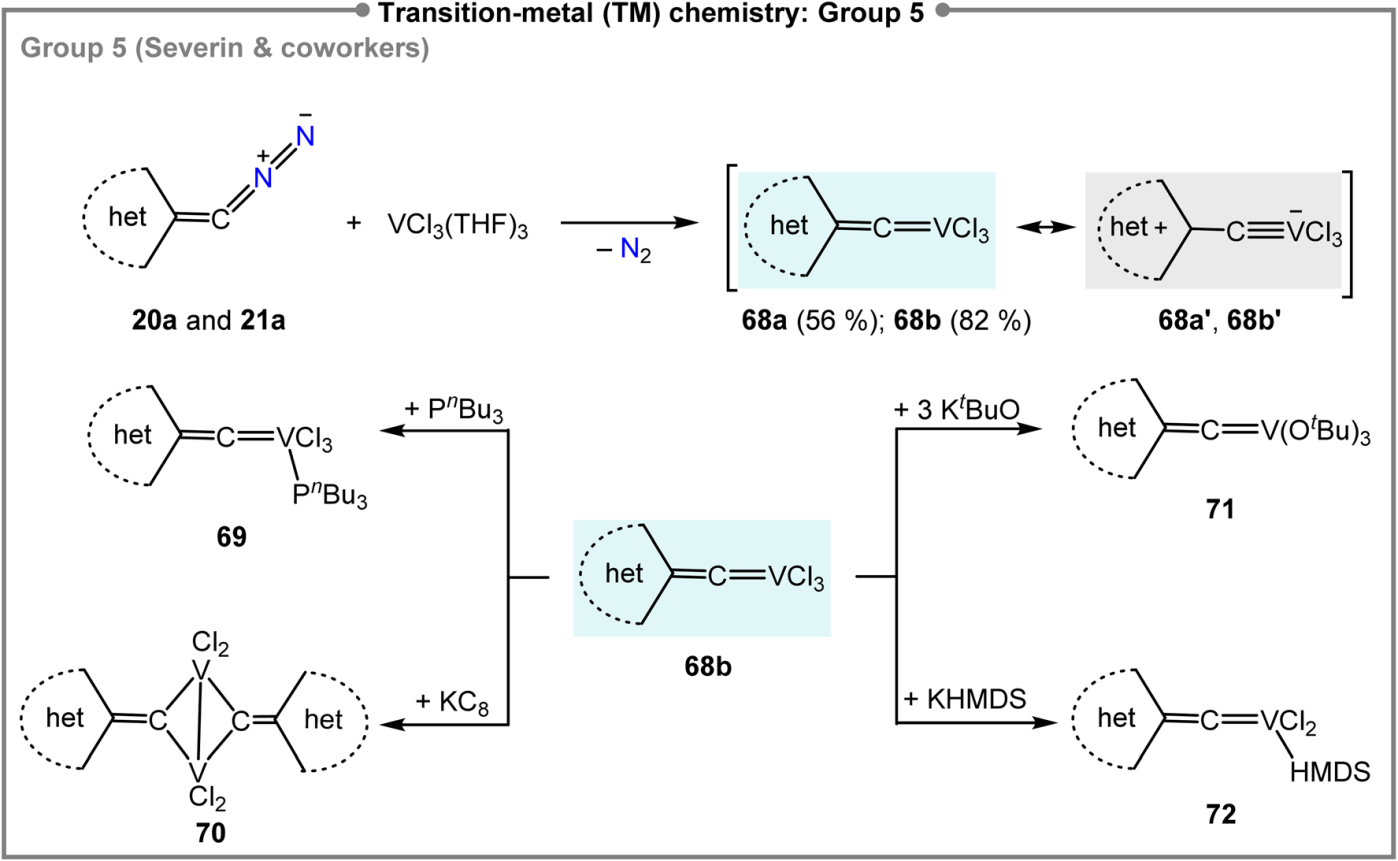
Synthesis of the vanadium vinylidene complexes 68a and 68b and the reactivity exploration of 68b.

“Free” NHVs have a triplet ground state,^130,131^ and by analogy to Schrock-type carbenes, which also feature alkylidene (63) ligands in a triplet-ground state,^132^ Severin and co-workers conceptualized that early TMs could be effective in stabilizing NHVs.^133^ The reaction of imidazole- and mesoionic-based diazoalkenes (20a and 21a; Fig. 2) with VCl_3_(THF)_3_ in Et_2_O at ambient temperature yields vinylidene complexes 68a and 68b, respectively (Fig. 11). This transformation proceeds through the facile liberation of N_2_. Single-crystal X-ray diffraction (scXRD) analysis of complexes 68a and 68b indicates that the NHV ligands coordinate to the vanadium centres in a nearly linear arrangement, exhibiting slightly shorter C–V bonds (68a: 1.706(2) Å; 68b: 1.704(2) Å), which suggests partial triple bond character. Density functional calculations for 68a and 68b indicate that the HOMO and HOMO−1 are primarily composed of two mutually perpendicular π-bonding orbitals, thereby supporting the significance of the 68a′ and 68b′ resonance forms (Fig. 11) and triple bond character.^133^ The initial reactivity analysis of 68b shows threefold substitution with K^*t*^BuO (71) and single substitution with KHMDS (72). The Lewis acidity of the vanadium centre in compound 68b is evidenced by its reaction with P^*n*^Bu_3_, resulting in the formation of compound 69. Subsequent reductive dehalogenation with KC_8_ generates the dinuclear complex 70, in which the vinylidene ligand functions as a µ^2^-bridging group (Fig. 11).

#### 4.4.3. Group 6

**Fig. 12:**
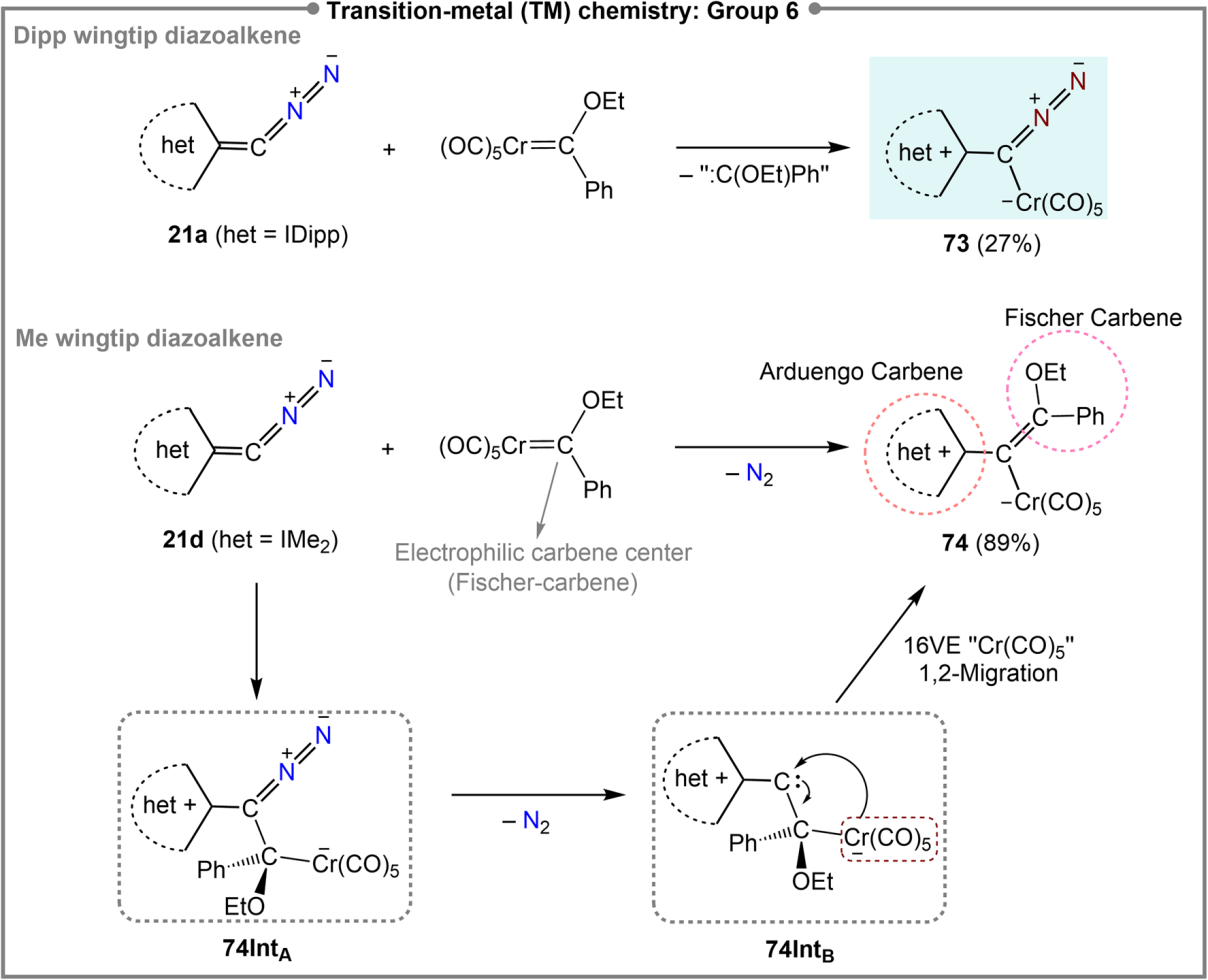
Group 6 reactivity of diazoalkenes 21a and 21d.

N-heterocyclic diazoalkenes possess an electron-rich vinylidene (C^VNL^) carbon centre and can readily coordinate to main-group- or transition-metal-based Lewis acids.^8,32^ Severin and co-workers reported that the diazoalkene bearing Dipp wingtip group (21a) reacts smoothly with the Fischer carbene complex (CO)_5_Cr=C(OEt)Ph, accompanied by substitution of the carbene fragment “:C(OEt)Ph”, to afford the corresponding Cr(CO)_5_-coordinated diazoalkene adduct 73 in 27% isolated yield (Fig. 12).^126^ This ligand-displacement reactivity is reminiscent of the reactions of NHCs with Fischer–carbene complexes.^134^ Notably, the diazoalkene bearing methyl substituents instead of Dipp wingtip groups (21d) undergoes a clean denitrogenative C–C coupling reaction, leading to the formation of a mixed Arduengo–Fischer-type carbodicarbene complex 74 coordinated to Cr(0) in excellent yield. Remarkably, the resulting complex exhibits appreciable stability toward air and moisture over several hours. Mechanistically, the formation of complex 74 likely proceeds through an initial nucleophilic attack of the diazoalkene 21d at the carbene carbon atom (C^carb^) of (CO)_5_Cr=C(OEt)Ph to generate intermediate 74Int_A_. Subsequent extrusion of N_2_ affords possible intermediate 74Int_B_, followed by migration of the 16VE “Cr(CO)_5_” fragment to furnish complex 74 (Fig. 12).^126^

#### 4.4.4. Group 9

**Fig. 13:**
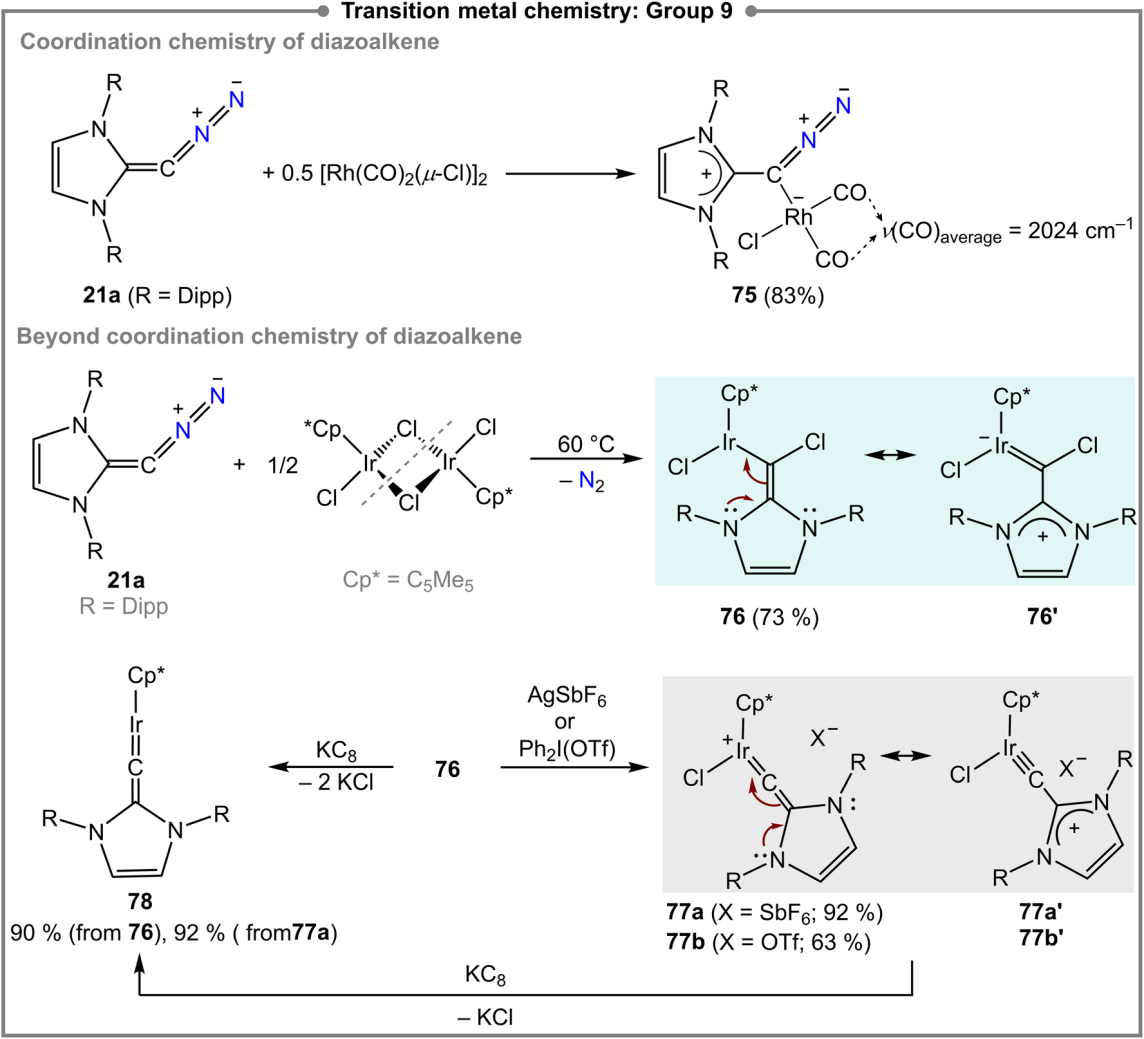
Group 9 reactivity of the diazoalkene 21a.

Diazoalkenes with a nucleophilic vinylidene carbon centre (C^VNL^) readily form stable, isolable adducts (75) with group 9 transition metals such as Rh(i), as demonstrated by Severin and co-workers (Fig. 13).^8^ Coordination of 21a to Lewis acidic metal complexes [Rh(CO)_2_Cl]_2_ is expected to strengthen the double ylidic resonance structure. This results in lengthening of the C^NHC^–C^VNL^N2 and C^VNL^–N_2_ bonds in 75. The IR spectrum of complex 75 displays strong absorption bands for *cis*-oriented CO ligands, with an average frequency of 2024 cm^−1^.^8^ This value shows that diazoolefin 21a is a stronger σ-donor than typical five-membered NHCs (*ν*(CO)_average_ = 2035–2046 cm^−1^).^135^

The strong electron-donating properties of the azolylidene fragment render NHVs effective *C*-donor ligands. The reaction of 21a with the dimeric Ir(iii) complex (Cp*IrCl_2_)_2_ at 60 °C affords the rare Ir-based halo alkylidene complex 76 as the major product (Fig. 13).^136^ The formation of 76 likely proceeds *via* C^VNL^ addition to the Ir-centre, generating a non-putative intermediate, followed by N_2_ elimination and subsequent 1,2-chloride migration from Ir to the C^VNL^ centre. The N-heterocyclic vinylidene (NHV) motif in 76 was unambiguously confirmed by scXRD analysis. Note that the structurally related phosphinidenide complex Cp*IrCl-(P=IDipp) was previously reported by Tamm *et al.*^137^ The C–Cl bond in compound 76 can be reversed by the addition of the halide scavenger AgSbF_6_ or Ph_2_I(OTf), resulting in the formation of the unprecedented cationic complexes 77a and 77b, which contain a terminal NHV motif and also possess significant Ir-based carbyne character (77a′ and 77b′) (Fig. 13). Reductive dehalogenation of compound 77a with three equivalents of KC_8_ yields vinylidene complex 78 (Fig. 13) in excellent yield. Alternatively, complex 78 can be accessed *via* the two-electron reduction of compound 76 with KC_8_. Single-crystal XRD analysis revealed that complex 78 adopts a distinctive ‘pogo stick’ geometry.^136^

#### 4.4.5. Group 10

**Fig. 14:**
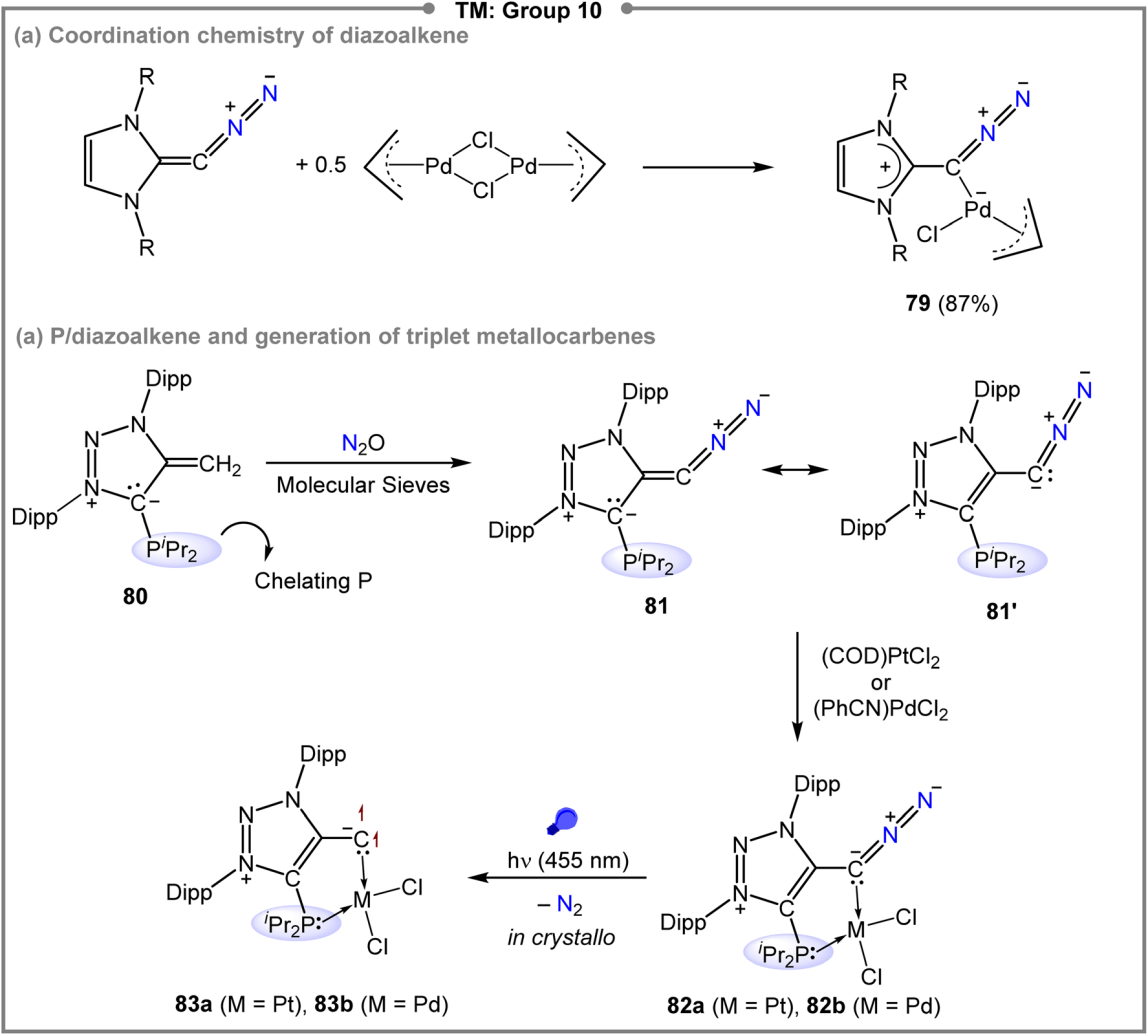
(a) Coordination chemistry of diazoalkene 21a with a Pd(i) metal complex; (b) synthesis of the P/diazoalkene 81 and generation of triplet metallocarbenes 83a and 83b.

Diazoalkenes with a nucleophilic vinylidene carbon centre (C^VNL^) readily form stable, isolable adduct (79) with group 10 transition metals such as Pd(ii), as demonstrated by Severin and co-workers (Fig. 14a).^8^

Triplet carbenes featuring a metal adjacent to the carbene centre (metallocarbenes; R–C–M) represent an emerging yet rarely explored class of *mono*-centred diradicals within the broader field of reactive intermediates.^138,139^ While singlet metallovinylidenes (R_2_C=C=M) are well known, the analogous triplet metallovinylidenes (R_2_C–C→M) are underrepresented.

To address this longstanding gap, we envisioned that a chelating P/diazoalkene precursor (81) could provide access to the desired triplet metallovinylidenes. Our group recently described the phosphorus-based chelating N-heterocyclic olefin (P-mNHO) (80), which undergoes diazo transfer in the presence of N_2_O to furnish the desired P/diazoalkene (81) in high yield (93%).^140^ In pursuit of accessing metallovinylidenes, compound 81 forms rigid P/C-chelating adducts with Pt(ii) and Pd(ii) centres, yielding complexes 82a (M = Pt) and 82b (M = Pd). The five-membered ring C/P-chelating ligand imposes geometric constraints on the R–C→M angle, effectively suppressing free bending at the carbene centre. Notably, irradiation of complexes 82a and 82b generates triplet metallovinylidenes 83a (M = Pt) and 83b (M = Pd). These species were unambiguously characterized at low temperature by THz-EPR spectroscopy and SQUID measurements, featuring large triplet zero-field splitting values of *D* = 124.5 cm^−1^ (Pt) and 8.0 cm^−1^ (Pd). Note that free triplet vinylidenes typically feature *D* values of 0.37–0.4 cm^−1^.^130,141^ Clear evidence for the formation of the desired triplet metallovinylidene was obtained through photochemically triggered *in crystallo* X-ray diffraction analysis (Fig. 14b).^140^

#### 4.4.6. Group 11

**Fig. 15:**
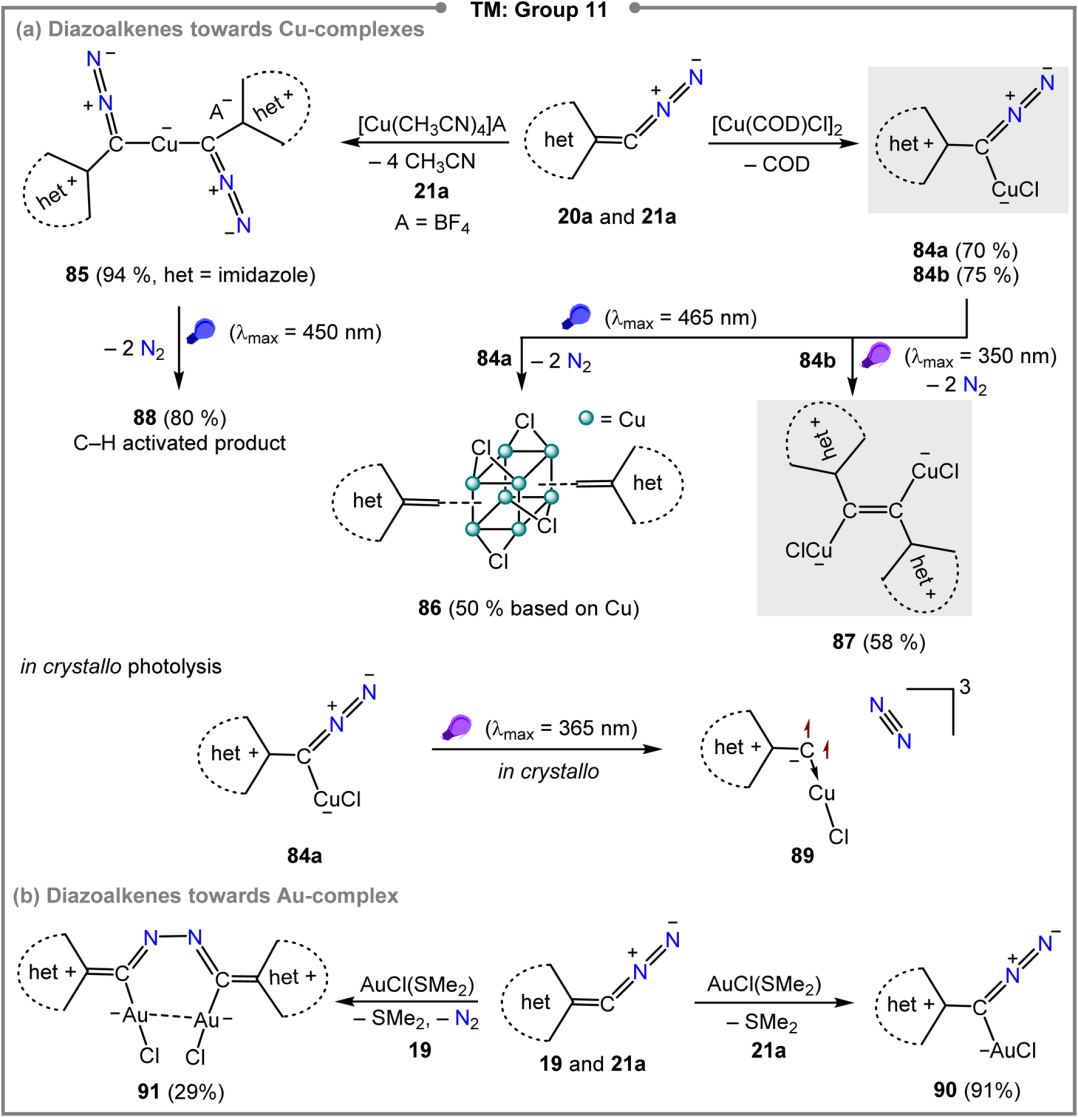
(a) Reactivity of diazoalkenes (20a and 21a) towards Cu-complexes and their photochemical reactivity; (b) reactivity of diazoalkenes (19 and 21a) towards an Au-complex.

The click reaction is a popular cyclization method that couples alkynes with diazonium betaines and 1,3-dipolar species such as RCNO, RN_3_, and R_2_CN_2_.^74^ The transformation proceeds *via* a µ-vinylidene intermediate.^142^ In the pursuit of accessing intermediates of these synthetically important transformations, Severin and co-workers treated the diazoalkenes (20a and 21a) with neutral-dimeric Cu(i)-complex [Cu(COD)Cl]_2_ and cationic [Cu(CH_3_CN)_4_]BF_4_ to afford stable and thermally robust adducts 84a, 84b and 85 (Fig. 15a).^143^ Irradiation of complex 84a at 465 nm resulted in the formation of the cubane-type Cu_8_ cluster 86. In contrast, irradiation of the analogous complex 84b at 350 nm generated the binuclear complex 87, which can be characterized as a two-fold Cu(i) chloride Lewis acid–base adduct of non-isolable (NHC)_2_C_2_.^144^ The homoleptic complex 85 loses molecular nitrogen upon irradiation (*λ*_max_ = 450 nm) to afford C–H inserted product 88 (Fig. 15a).^143^

The isolation of the photo-irradiated species discussed provides strong evidence that the transient vinylidene functions as a reactive intermediate. To achieve this isolation, Severin and Powers *et al.* conducted *in-crystallo* photolysis^145^ of 84a. Exposure of a single crystal of 84a to irradiation (λ_max_ = 365 nm) at 100 K resulted in its conversion to 89 and N_2_ within the crystal lattice (Fig. 15a). Density functional theory (DFT) calculations show that the alkenylidene complex 89 possesses a triplet ground state 89,^3^ which is separated by approximately 50.4 kJ mol^−1^ from the lowest singlet state 89.^1,143^ Interestingly, while the *in-crystallo* X-ray diffraction indicated a bent structure [∢C–C–Cu = 138.1(2)°], computations predicted a linear minimum structure on a fairly flat potential energy surface. This discrepancy might be due to packing effects or the inability to fully relax into the minimum structure in the solid-state lattice.

Additionally, Dipp wingtip-based diazoalkene 21a exhibits exclusive coordination chemistry with an Au(i)-based Lewis acid, resulting in the formation of stable Lewis acid–base adduct 90 with an excellent yield of 91% (Fig. 15b).^8^ In contrast, the mesoionic-carbene-based diazoalkene 19 produces the dimerization product 91 in a low, isolable yield. The aurophilic Au⋯Au interaction favours the *s-cis* configuration over the *s-trans* configuration in 91 (Fig. 15b).^32^

### 4.5. Beyond diazoalkenes: neutral-cumulenic diazo compounds – surrogate of atomic carbon and CN_2_ fragments

**Fig. 16:**
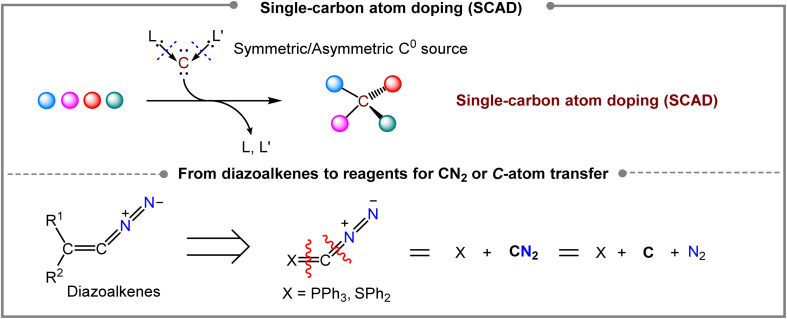
A general representation of single carbon atom doping (SCAD) from a/symmetric ylides.

Inserting an atomic carbon into a reactant molecule without losing atoms from the reactant is referred to as Single-Carbon Atom Doping (SCAD, Fig. 16).^146–148^ Electric discharge of a carbon arc, followed by vaporization into a low-temperature matrix, is the common method for generating atomic carbon equivalents.^149–151^ However, such a species is highly reactive and requires a sophisticated setup and careful control of the reaction conditions. Often such an approach is severely limited to structurally simple compounds with low yields of C-atom transfer products. Development of new, stable reagents to reduce reactivity and enable easier laboratory handling is still in its infancy.^152^ One of the simplest ways to represent a convenient source for atomic carbon is its zerovalent C^0^ form (typically called carbones/bis-ylides), supported by two neutral 2e donor ligands (Fig. 16).

Isolable symmetric X,X-bis(ylides) (X = CR_2_, PR_3_, SR_2_) and asymmetric X,Y-bis(ylides) (X = CR_2_, PR_3_; Y = PR_3_, SR_2_) have been reported and their geometric properties associated with their coordination chemistry have been studied extensively.^153^ However, their chemical application as an atomic carbon surrogate has largely remained unexplored, with the exception of P,S-bis(ylide) R_3_P=C=SR_2_.^154^ Baceiredo and co-workers reported that an asymmetric P,S-bis(ylide) could act as a carbon source in a multicomponent reaction.^154^ In theory, CDPs could conceivably react with two equivalents of ketones to form cumulenes *via* a double-Wittig reaction. However, direct proof of these observations is lacking.^155^ The limited exploration of asymmetric ylides as potent carbon atom surrogates could be attributed to the non-facile cleavage of supported ligands.

**Fig. 17:**
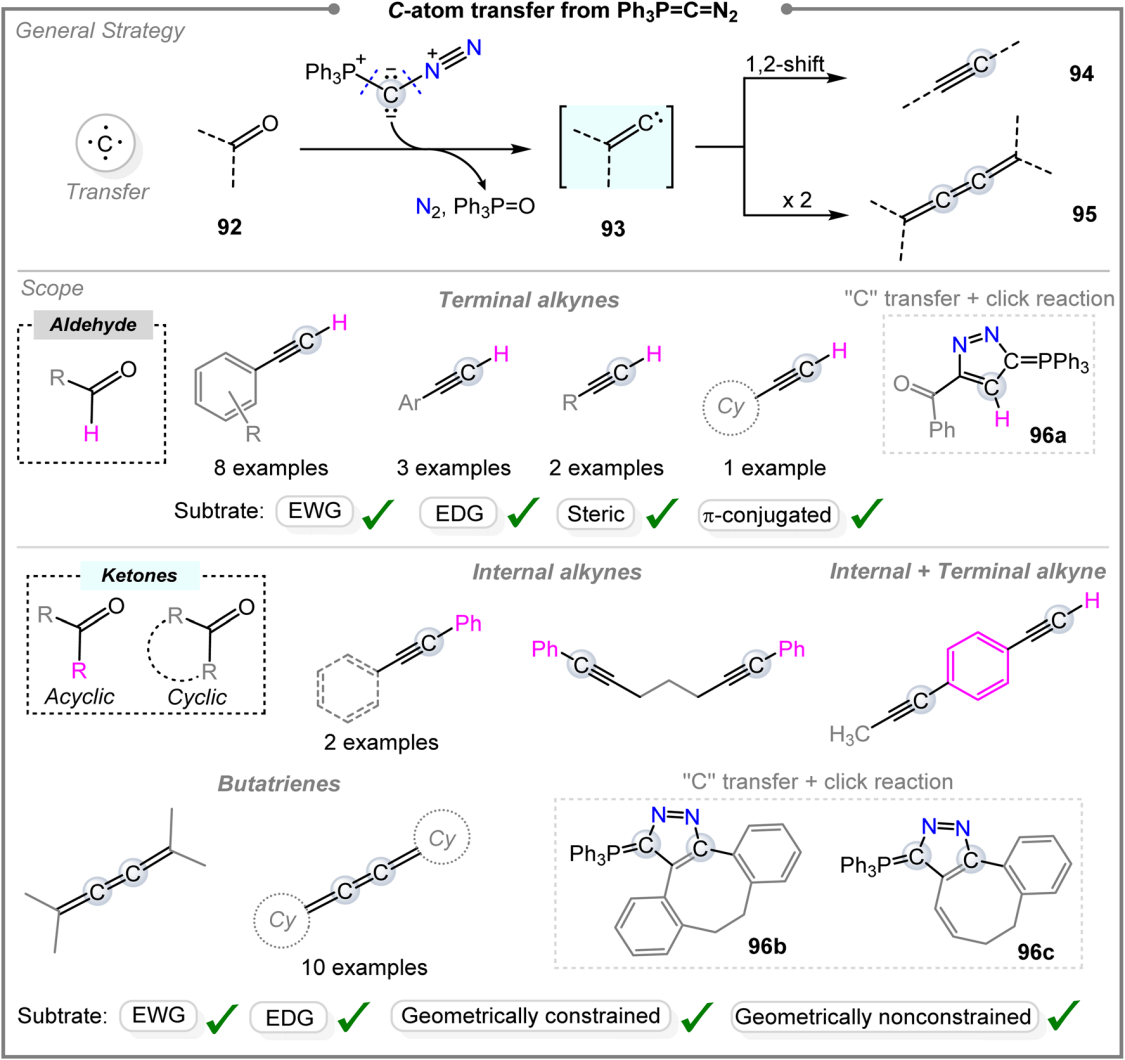
“C(sp)”-atom transfer reactivity of diazophosphorus ylide 29a.

The challenge of employing carbones as atomic carbon surrogates prompted the conceptualization of a C^0^ atom flanked by two simple, labile groups, one of which is N_2_, as an ideal reagent for organic transformations. We hence reasoned that the exchange of the R_2_C fragment in diazoalkenes with P- or S-ylides should enable the development of potent reagents for the transfer of CN_2_ moieties or C atoms. In the case of P-ylides, Bertrand *et al.* reported in 1987 the isolation of the pseudo-unsaturated diazo compound (^*i*^Pr_2_N)_2_ClP=C=N_2_ (ref. 44), which made it realistic to target the access of the unknown R_3_P=C=N_2_ compound as a potential surrogate for atomic carbon. Based on the N_2_O activation strategy ([3 + 2] followed by the retro-[3 + 2]) outlined above, we successfully isolated the first thermally robust and crystalline neutral-cumulenic diazo compound Ph_3_P=C=N_2_ (29a).^40^ The reaction between aldehydes (92a–g, Fig. 17) and 29a exclusively produces the corresponding alkynes, hence transferring a C(sp)-atom. The process involves a Wittig reaction that generates transient and highly reactive diazoalkenes, subsequent N_2_ loss forming vinylidene, and a final 1,2 H shift. Aryl aldehydes (ArCHO) and alkyl aldehydes (RCHO) possessing diverse electronic properties, functional groups, and steric environments afforded alkynes in high yields (Fig. 17). α-Ketoaldehyde phenyl glyoxal selectively yields phosphoranylidene pyrazole 96a*via* a one-pot “C-atom” transfer in combination with a click reaction of another equivalent of Ph_3_PCN_2_. In contrast, efficient “C-atom” transfer from diazophosphorus ylide 29a necessitates a more electron-deficient carbonyl carbon, as demonstrated by the absence of reactivity between 29a and acyclic amides or esters, even at elevated temperatures.^40^

Using dialkyl or cyclic ketones as substrates reduces the likelihood that substituents undergo a 1,2-shift in the vinylidene intermediate (93), thereby facilitating the isolation of the corresponding butatrienes (95). For instance, acetone, the simplest ketone, led to the selective formation of tetramethylbutatriene, with no detectable internal alkyne (Fig. 17). Various cyclic ketones also led to the selective formation of butatrienes. Cyclic arylketones with reduced geometric constraints facilitate the synthesis of phosphoranylidene pyrazoles 96b and 96c, thereby enabling a one-pot preparation of fused aromatic heterocycles from simple ketones. Experimental results indicate that less-strained cyclic ketones preferentially undergo the 1,2-shift rather than dimerization, thereby supporting the formation of geometrically strained alkynes through “C”-atom transfer to a cyclic ketone.^40^

**Fig. 18:**
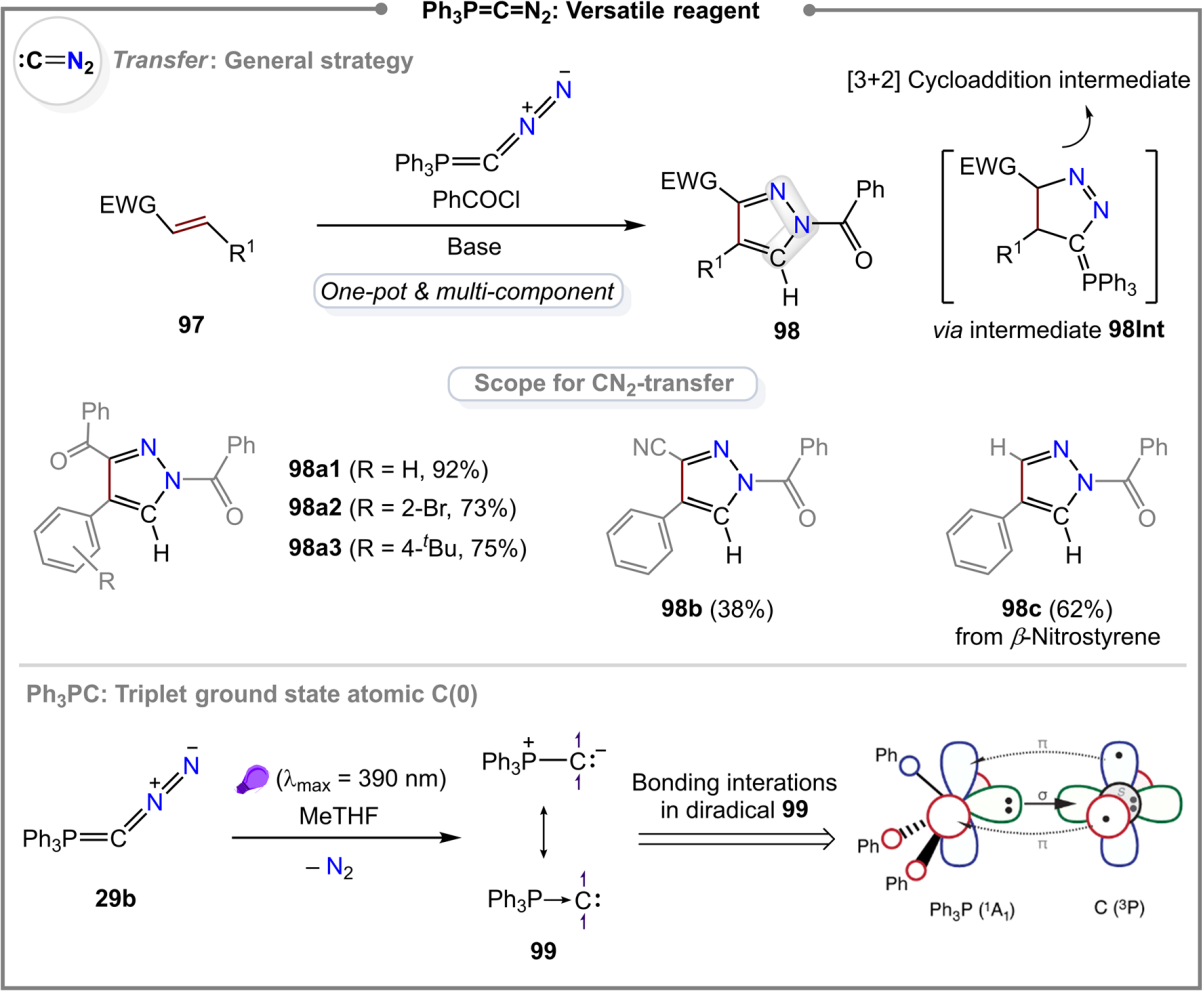
(Top) accessibility of highly substituted pyrazoles (98a–c) *via* CN_2_ group transfer from diazophosphorus ylide 29b to electron-deficient alkenes; (bottom) irradiation-induced N_2_ extrusion from 29b at cryogenic temperatures generating monosubstituted Ph_3_P → C (99) in its triplet ground state.

Additionally, diazophosphorus ylide 29b serves as a CN_2_ fragment donor to electron-deficient alkenes in a highly chemoselective fashion. Its reaction with *trans*-chalcones (97) undergoes a formal [3 + 2] cycloaddition, affording the transient phosphoranylidene pyrazole 98Int. Subsequent proton transfer, electrophilic functionalization with PhCOCl, and PPh_3_ elimination under basic conditions (NaH) furnish highly substituted pyrazoles (98a1–98c) in good to excellent yields (Fig. 18a). α,β-Unsaturated alkenes, including *trans*-chalcones bearing diverse functionalities (98a1–a3) and cyanoalkenes (98b), were well tolerated under the reaction conditions. In the case of β-nitrostyrene, denitrative elimination of the NO_2_ group was also observed, enabling the synthesis of pyrazole 98c, lacking an EWG on the pyrazole scaffold (Fig. 18).^40^

Over the last two decades, diradicals have garnered significant attention, establishing a broad platform for the isolation of reactive intermediates and simultaneously expanding their applications in photophysical properties.^156,157^ However, monovalent carbon-centred diradicals remain largely underexplored, with only a report from our group on triplet vinylidenes.^130,141^ A key limiting factor is the lack of suitable synthons capable of addressing these longstanding gaps in C-centred reactive intermediate chemistry. Conceptually, diazophosphorus ylide 29b bearing a labile N_2_ unit offers a promising entry point to address this long-standing gap. Upon UV irradiation at cryogenic temperatures in a frozen MeTHF matrix, 29b undergoes photochemical N_2_ extrusion to furnish the monophosphine-supported atomic carbon species Ph_3_P→C (99; Fig. 18).^158^ The resulting diradical 99 was characterized by EPR spectroscopy in conjunction with isotope-sensitive ENDOR measurements at low temperatures. Notably, Ph_3_P→C displays an axial zero-field splitting parameter of *D* = 0.543 cm^−1^ with an exceptionally small rhombicity (|*E*|/*D* = 0.002), indicative of a highly axial electronic environment. Time- and temperature-dependent studies further confirm a triplet ground state, with a lifetime of approximately 10 min at 127 K in toluene-d_8_. In-depth computational analysis suggests that the bonding in Ph_3_P→C (99) can be rationalized by the direct involvement of carbon in its ground-state ^3^P term, arising from the 2s^2^2p^2^ electronic configuration and donation of PPh_3_ in its singlet symmetric ground state ^1^A_1_ (Fig. 18).^158^

Within the context of skeletal editing,^159^ carbon insertion involves the introduction of “C–R” fragments into C(sp^2^)–C(sp^2^) bonds, which typically produces planar, aromatic (hetero)cyclic structures.^146,147^ Pure carbon-atom transfer reactions entail the movement of a single carbon atom, exemplified by the Seyferth–Gilbert homologation, the Corey–Fuchs reaction,^160^ the Doering–LaFlamme allene synthesis,^161^ and the Ph_3_PCN_2_ (29a) reagent developed by our research group. These methods proceed *via* an unsaturated carbene or vinylidene intermediate, ultimately generating sp-hybridized carbon atoms. Consequently, the formation of 3D structures using these reagents is not feasible. We recently reported the synthesis and isolation of the stable crystalline diazosulfur ylide Ph_2_S=C=N_2_ (29b), utilizing it for a longstanding challenge of C(sp^3^)-atom transfer.^41^

**Fig. 19:**
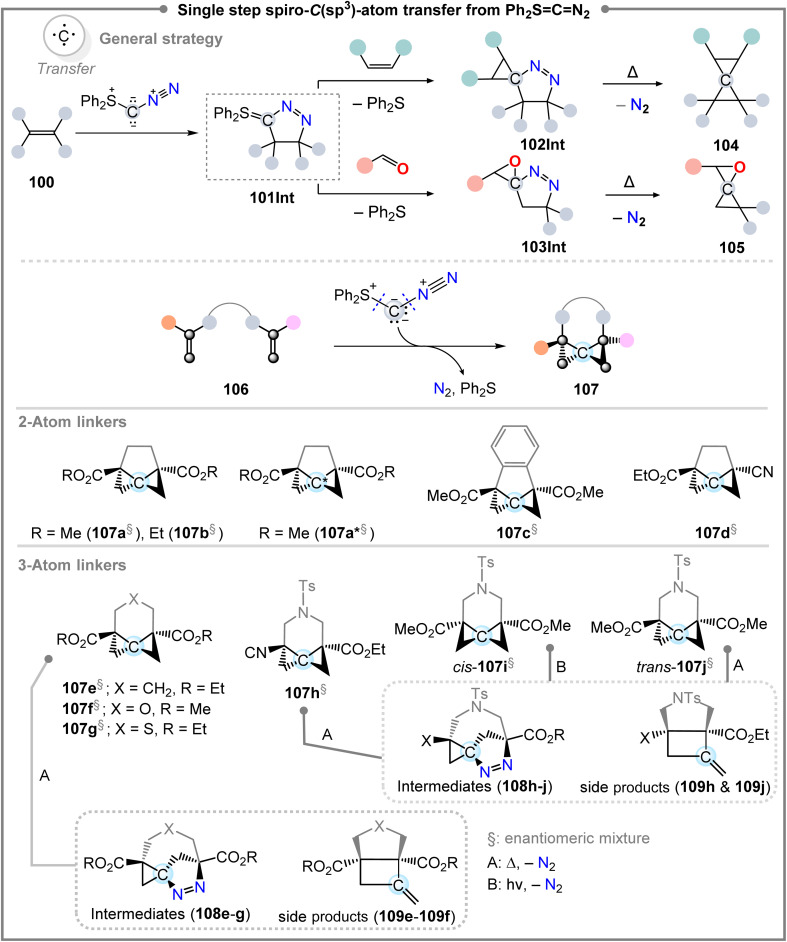
Single step spiro-C(sp^3^)-atom transfer from diazosulfur ylide 29b to afford bridged spiropentanes.

Instead of two-step ligand dissociation and direct C-atom transfer, a more rational reaction sequence was envisioned: [3 + 2] cycloaddition of Ph_2_S=C=N_2_ (29b) with acceptor substituted alkenes results in 101Int, which features a sulphur ylide moiety. Corey–Chaykovsky cyclopropanation (102Int) or epoxidation (103Int) followed by thermal N_2_ elimination leads to either spiro[2.2]pentane motifs (104) or oxaspiro[2.2]pentanes (105) (Fig. 19). Based on these results, it was hypothesized that acceptor-group-substituted 1,5-hexadienes (106a–d) could function as suitable substrates, facilitating both cycloaddition and cyclopropanation within a single molecule. Notably, the reaction of 29b with either dimethyl or diethyl 2,5-dimethylenehexanedioate yields the complex tricyclic spiro-pentanes 107a and 107b in a single step. To demonstrate proof of concept, the reaction was also conducted with ^13^C-labeled 29b-^13^C, resulting in the selective formation of 107a*. The *o*-phenyl-bridged dienes (106c) afford tetracyclic systems (107c). The newly generated spiro-carbon atom forms four new C–C bonds and adopts a highly distorted seesaw–disphenoidal geometry. The annulated C–C bond is elongated to alleviate ring strain. The C-atom transfer methodology is also applicable to the unsymmetrical two-atom linker substrate 106d, yielding the ester/nitrile-functionalized tricycle 107d. All saturated tricycles in 107a–107d series exhibit stability at room temperature, with the exception of 107c, which undergoes gradual degradation in solution as a consequence of ring strain (Fig. 19).^41^

To further evaluate the substrate tolerance of 29b, variations in ring size and heteroatom incorporation were investigated (Fig. 19). Application of an extended three-atom tether, consisting of CH_2_, O, and S moieties, enabled efficient synthesis of isolable intermediates (108e–108g) in high yield. Subsequent heating of these intermediates resulted in N_2_ liberation and formation of the target bridged spirotricycles (107e–107g) as the predominant products, while minor side products (109e and 109f) were detected in low concentrations. The reaction of the asymmetric diene yielded tricycle 107h, with formal [3 + 2] cycloaddition occurring at the ester-substituted olefin and cyclopropanation at the nitrile-substituted olefin. The incorporation of an *N*-tosyl bridge facilitated the isolation and scale-up of intermediate tricycle 108j, which subsequently degrades into target tricycles. The identity of the resulting tricycle is determined by the degradation pathway. Under thermal conditions, *trans*-tricycle 107j is produced in low yield (15%), with 108j remaining the predominant product. In contrast, irradiation with an LED at *λ*_max_ = 370 nm yields *cis*-tricycle 107i as the major product (*cis* : *trans* = 9 : 1) (Fig. 19). Calculations indicate that the *cis*-tricyclo[5.1.0.0 (ref. 1 and 3)]octane core possesses higher strain energy than the *trans*-isomer (Δ*E*_*cis*−*trans*_ = 31 kcal mol^−1^) and demonstrates the most significant structural deformation (twisting), among the spiro-cyclopentanes reported in the CCDC structural database.^41^

### 4.6. Neutral-ligand exchange at Ph_2_SCN_2_: access to crystalline Ph_2_SCCO as a masked ketenylidene source

**Fig. 20:**
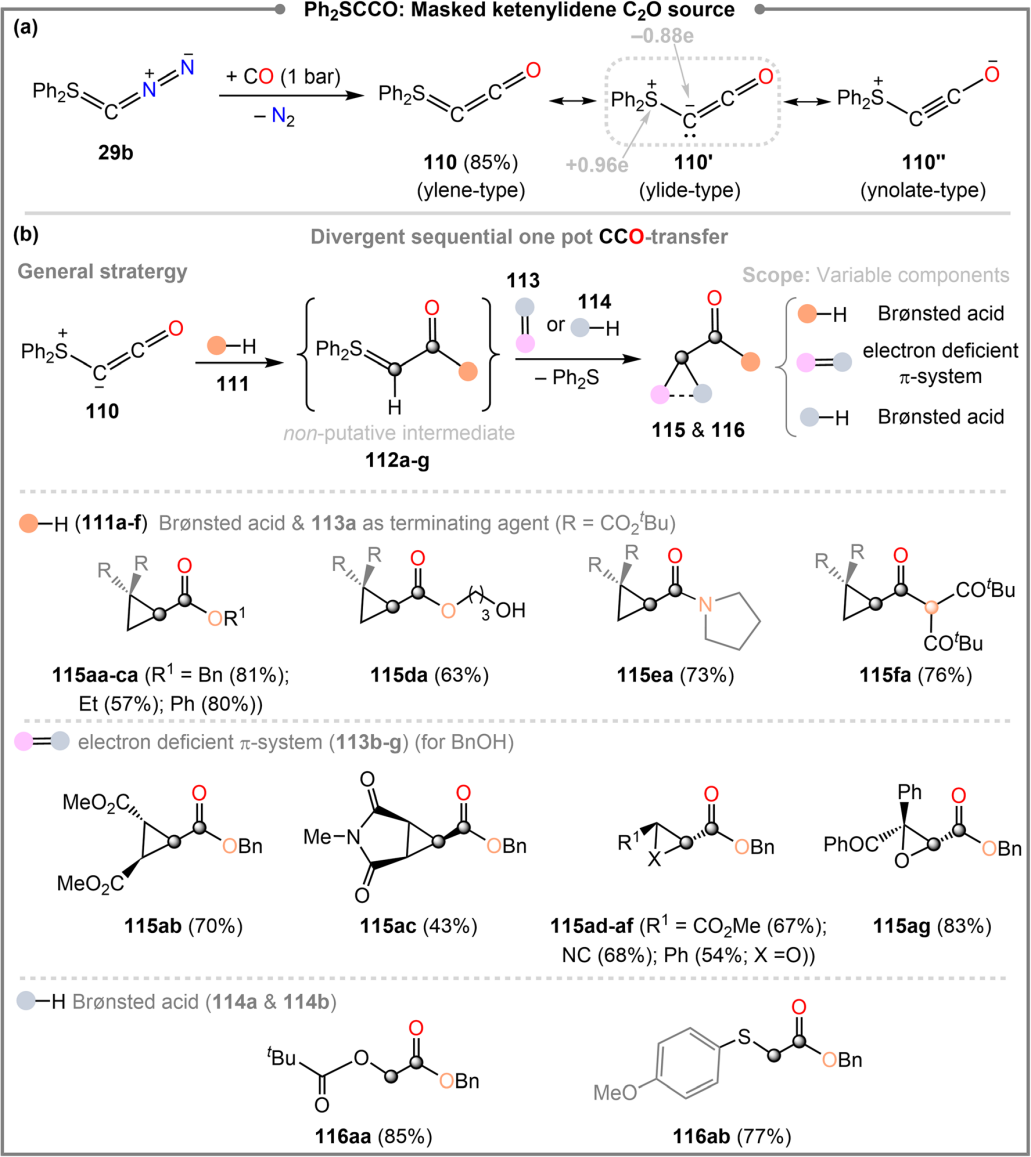
(a) Synthesis of S-based heterocumulene 110; (b) reactivity study of 110 as a divergent sequential one-pot CCO-transfer reagent.

The previously described strategy of formal N_2_/CO exchange from neutral-cumulenic diazo compounds X=C=N_2_ (X = NHC, PPh_3_) can be applied to diazo sulfur ylide 29b, which undergoes a selective N_2_/CO exchange to yield the unprecedented colourless crystalline S-based heterocumulene (ketene) Ph_2_SCCO (110, Fig. 20a) with an excellent yield of 86%.^162^ Classical ketene synthesis strategies, such as base-induced 1,2-elimination from esters,^163,164^ as applied in the well-documented Bestmann's ylide Ph_3_PCCO,^165^ did not yield any positive outcome for 110 and necessitated the formal N_2_/CO exchange pathway to afford anticipated highly reactive S-based heterocumulene 110. Structural analysis of 110 using single-crystal X-ray diffraction (scXRD) and density functional theory (DFT) calculations indicates that ylide-type 110′ is the predominant natural Lewis structure, rather than ylene 110 or ynolate 110″. Natural population analysis (NPA) at the PBE0-D3(BJ)/def2-TZVP level shows a full negative charge on C1 (−0.88*e*) and a fully positive charge on the adjacent S-atom (+0.96*e*). C2 and the O atom display the typical characteristics of a polarised C=O bond. The pronounced localisation of electron density at C1 suggests that 110 should exhibit nucleophilicity and Brønsted basicity at the core carbon.^162^

As anticipated, the S-based heterocumulene 110 undergoes a facile 1,2-addition across the C=C double bond in the presence of Brønsted acids (111a–g), affording ester-derived S-ylide intermediates (112a–g) with varying stability. Notably, the *in situ*-generated S-ylides (112a–g) participate in classical Corey–Chaykovsky-type reactivity with polarized alkenes, as well as substrates bearing polar C=O functionalities, and also undergo non-metal mediated insertion into polar X–H bonds, leading to cyclopropanation, epoxidation, and the formation of hetero-substituted acyclic esters as CCO transfer products (Fig. 20b).^162^

In general, for the CCO-transfer strategy, a variety of alcohols (111a–d) were evaluated using the same Michael acceptor, 113a (di-*tert*-butyl methylenemalonate), as the terminating reagent in a one-pot synthesis to furnish α-cyclopropyl esters (115aa–da). Although the use of strongly acidic components (p*K*_a_ ≈ 10 – 16) to generate S-ylides in the initial step raises questions regarding the generality of the CCO-transfer protocol, the method proved unexpectedly compatible, even with non-acceptor substituted amines (*e.g.*, pyrrolidine, 111e), affording α-cyclopropylamide 115ea in 73% isolated yield. Notably, even C–H acidic compounds such as dipivaloylmethane (111f) were tolerated, forming three new C–C bonds to yield cyclopropyl ketone 115af (Fig. 20b).^162^ The terminating agent (polarized alkene, 113a–g) was varied to further explore the substrate scope of the transformation. Ester-derived S-ylides (112a–g) generated from benzyl alcohol (111a) (BnOH) underwent smooth reaction with dimethyl fumarate (113b) to provide the corresponding 1,2,3-substituted cyclopropane 115ab. Likewise, reaction with the cyclic 1,2-disubstituted olefin *N*-methylmaleimide (113c) furnished the azabicyclo[3.1.0]hexane derivative 115ac in a moderate yield of 43%. In addition, S-ylides reacted effectively with mono-acceptor-substituted olefins, including methyl acrylate (113d) and acrylonitrile (113e), delivering the corresponding disubstituted cyclopropanes 115ad and 115ae. S-Ylides are well recognized for their ability to transform aldehydes and ketones into the corresponding epoxides.^166,167^ To validate the reactivity of the generated ester-based S-ylides, reactions with benzaldehyde (113f) and benzil (113g) were carried out, affording the corresponding epoxides 115af and 115ag, respectively. Finally, the *in situ* generated ester-based S-ylides underwent transition-metal-free formal insertion into polarized X–H bonds. For example, reactions with the highly acidic pivalic acid (116a) and *N*-methoxythiophenol (116b) furnished the corresponding pivalate ester 116aa and thioether 116ab, respectively, in high yield (Fig. 20b).^162^

## 5. Conclusion and outlook

New classes of compounds frequently emerge from scientific curiosity or unanticipated laboratory observations, although their practical applications may require decades to materialize. This review discusses the isolation, characterization, and distinctive reactivity of neutral-cumulenic diazo compounds. These compounds have progressed from transient laboratory curiosities and non-isolable intermediates to a stable substance class with significant and diverse applications across all element groups in the periodic table. Recent advances in cumulenic diazo compound chemistry, particularly through the isolation of NHCs and mNHC/aNHC-based diazoalkenes, have yielded more reactive cumulenic diazo compounds with considerable synthetic potential. NHCs and mNHC/aNHC-based diazoalkenes enable the synthesis of previously unreported main group compounds from Groups 13, 14, and 15, which remain inaccessible using conventional precursors. Most of the chemistry has so far only been developed using NHC derived diazoalkenes, but we believe that the other recently emerging neutral-cumulenic diazo compounds either bearing different heterocycles or P/S-ylides hold very high potential for applications throughout the elements in the periodic table. The isolated BN-embedded heterocumulene exhibits promising applications in materials chemistry. Additionally, diazoalkenes function as effective precursors for metal complexes containing NHV ligands. Although NHV generally serves as a 2e^−^ (2σ) or 4e^−^ (2σ + 2π) donor in its neutral resonance form, the vanadium and iridium complexes presented in this study demonstrate NHVs acting as 6e^−^ donors (2σ + 4π) in their dianionic resonance form, thereby rendering them analogous to imido (RN^2−^) type ligands. The isolable, thermally stable diazophosphorus ylide enables a direct one-step carbon atom transfer reaction, characterized by thermal stability and broad substrate compatibility. Diazosulphur ylide Ph_2_S=CN_2_ functions as a versatile C(sp^3^)-atom transfer reagent, facilitating either stepwise or single-step formation of up to four new C–C bonds around a highly strained spiro-carbon atom derived from an accessible two-dimensional feedstock. We believe that the coming years will spark a vibrant revival in synthetic main-group, transition-metal, and SCAD chemistry, fuelled by the exciting rise of neutral-cumulenic diazo compounds.

### 5.1. Broader outlook

The emergence of boron, silicon, other main-group and transition metal-based diazo compounds as well as neutral/anionic cumulenic CN_2_-motif containing species marks a significant shift in the conceptual foundations of traditional diazo chemistry. The boron-based diazo compounds of the type (NHC)(R)BN_2_,^168,169^ recently reported by the groups of Gilliard and Cummins, should be highlighted, as these species can be considered isolobal to the stable diazoalkenes summarized in this work. This is especially intriguing because their stabilization enables direct observation and controlled reactivity, while still preserving the characteristic propensity for dinitrogen extrusion and small-molecule transfer.^170^ These findings indicate that the diazo motif is not inherently linked to carbon but instead represents a transferable electronic architecture that can be incorporated into electron-deficient main-group environments. Concurrently, donor–acceptor and “hidden-valence” bonding models, as advanced by Frenking and co-workers in the context of “C(0)” chemistry, provide a crucial theoretical framework for interpreting these compounds, particularly where conventional octet-based Lewis structures are inadequate.^39^ Additionally, significant are the Pt-supported CN_2_^2−^ and CN_2_˙^−^ species reported by Schneider and co-workers, in which doubly deprotonated diazomethane ligands were stabilised by Pt(II) pincer fragments to generate isolable terminal and bridging Pt–N=N=C–Pt motifs. These studies represent one of the clearest demonstrations that CN_2_ fragments can behave as electronically non-innocent ligands analogous to azides or nitrenes rather than merely transient intermediates.^171^ Maron, Mazzanti and co-workers further demonstrated the applicability of the CN_2_ motif in *f*-block actinide chemistry, thereby leading to the formation of an unusual Th^IV^ amido acetylido complex *via* abnormal cleavage of the imidazole heterocyclic backbone.^172^ Beyond the broader chemistry of neutral-cumulenic diazo compounds discussed in this perspective, diazo chemistry is evolving from a narrow focus on functional-group transformations to a comprehensive field of element-centred dinitrogen chemistry. In this respect, it might even be imaginable that such compounds could be accessed from dinitrogen fixation, again a highly active research field within main group chemistry.^173^ Despite significant progress in the synthesis and stabilization of diazoalkenes, several key challenges remain unresolved. In particular, the substrate scope of currently available systems remains relatively limited, with most stabilised diazoalkenes relying on strongly polarized substituents. This bias has hindered access to “genuine” diazoalkene motifs, which are expected to exhibit distinct electronic structures and reactivity patterns. In addition, catalytic applications of diazoalkenes remain largely unexplored, representing a significant opportunity for future development. Similarly, their potential photochemical reactivity has not been systematically investigated, despite the likelihood of rich excited-state behaviour. Beyond molecular reactivity, opportunities may also exist in materials science and small-molecule activation, where the unique bonding and electronic characteristics of diazoalkenes could be exploited. Addressing these limitations will require the development of more general stabilization strategies, as well as deeper mechanistic understanding of their bonding situation. Overall, these challenges highlight important directions for future research and underscore the untapped potential of diazoalkene chemistry.

## Conflicts of interest

The author declares no conflicts of interest.

## Data Availability

No primary research results, software, or code have been included, and no new data were generated or analyzed as part of this perspective.
